# Single-particle diffusional fingerprinting: A machine-learning framework for quantitative analysis of heterogeneous diffusion

**DOI:** 10.1073/pnas.2104624118

**Published:** 2021-07-28

**Authors:** Henrik D. Pinholt, Søren S.-R. Bohr, Josephine F. Iversen, Wouter Boomsma, Nikos S. Hatzakis

**Affiliations:** ^a^Department of Chemistry, University of Copenhagen, 2100 Copenhagen, Denmark;; ^b^Nanoscience Center, University of Copenhagen, 2100 Copenhagen, Denmark;; ^c^Department of Computer Science, University of Copenhagen, 2100 Copenhagen, Denmark;; ^d^Novo Nordisk Foundation Centre for Protein Research, Faculty of Health and Medical Sciences, University of Copenhagen, 2200 Copenhagen, Denmark

**Keywords:** fingerprinting, single-particle tracking, machine learning, fluorescence microscopy, stochastic processes

## Abstract

Single-particle tracking (SPT) is a key tool for quantitative analysis of dynamic biological processes and has provided unprecedented insights into a wide range of systems such as receptor localization, enzyme propulsion, bacteria motility, and drug nanocarrier delivery. The inherently complex diffusion in such biological systems can vary drastically both in time and across systems, consequently imposing considerable analytical challenges, and currently requires an a priori knowledge of the system. Here we introduce a method for SPT data analysis, processing, and classification, which we term “diffusional fingerprinting.” This method allows for dissecting the features that underlie diffusional behavior and establishing molecular identity, regardless of the underlying diffusion type. The method operates by isolating 17 descriptive features for each observed motion trajectory and generating a diffusional map of all features for each type of particle. Precise classification of the diffusing particle identity is then obtained by training a simple logistic regression model. A linear discriminant analysis generates a feature ranking that outputs the main differences among diffusional features, providing key mechanistic insights. Fingerprinting operates by both training on and predicting experimental data, without the need for pretraining on simulated data. We found this approach to work across a wide range of simulated and experimentally diverse systems, such as tracked lipases on fat substrates, transcription factors diffusing in cells, and nanoparticles diffusing in mucus. This flexibility ultimately supports diffusional fingerprinting’s utility as a universal paradigm for SPT diffusional analysis and prediction.

## Significance

Single-particle tracking (SPT) analysis of individual biomolecules is an indispensable tool for extracting quantitative information from dynamic biological processes, but often requires some a priori knowledge of the system. Here we present “single-particle diffusional fingerprinting,” a more general approach for extraction of diffusional patterns in SPT independently of the biological system. This method extracts a set of descriptive features for each SPT trajectory, which are ranked upon classification to yield mechanistic insights for the species under comparison. We demonstrate its capacity to yield a dictionary of diffusional traits across multiple systems (e.g., lipases hydrolyzing fat, transcription factors diffusing in cells, and nanoparticles in mucus), supporting its use on multiple biological phenomena (e.g., drug delivery, receptor dynamics, and virology).

Single-particle tracking (SPT) has enabled the quantitative analysis of dynamic biological processes with nanometer spatial and millisecond temporal resolution, revealing dynamic behaviors previously masked in ensemble averaging (1, 2). By direct detection and spatiotemporal localization of biomolecules, SPT provides molecular trajectories for dynamic biological processes with nanometer spatial and millisecond temporal resolution. These trajectories have offered key insights into receptor dynamics (3), clathrin-mediated endocytosis (4), molecular motors (5), transcription factor motion (6), viral entry (7), and efficient drug delivery (8). More generally, they have offered new insights into the complex interplay between the structure, function, and environment of biomolecules through the characteristics of their diffusion.

The characteristics of diffusion often correlate with functional traits of interest. For example, enzyme diffusion might increase with catalysis (9); G-protein–coupled receptors display altered diffusion upon ligand binding (3) or dimerization (10); and nanoparticle coatings alter drug-delivery efficiencies that are measurable as changed diffusion (11, 12). Single-particle tracking thus holds promise as a source of diffusional data for future advanced screening studies in a broad range of systems (13–16).

The rich information inherent in SPT data imposes direct analytical challenges: Biological motion is highly heterogeneous and displays a variety of diffusion types that may vary drastically across both systems and time and are dependent on regulatory cues or spatial localization, as we and others have shown (17–20). Dealing with such heterogeneity is challenging, as there is no one-model-fits-all solution. Depending on the phenomenon under investigation, most groups have developed their own methodologies for estimating both the diffusion type and the parameters of specific diffusion models analytically (21–29) or using machine learning (30–37). If the motion changes over the course of a trajectory, tools have also been developed to segment the trace into regimes that are consistent with a model of interest (35–41). These methodologies rely on identifying or comparing against a specific type of diffusion model and thus are not general, but rather are dependent on the complex phenomenon under investigation.

Here we address the challenge of providing a general method for SPT analysis, processing, and classification by implementing a diffusional fingerprint: a unique identifier for each observed SPT particle that allows for easy comparisons and precise entity prediction. Fingerprinting has been employed in fields as diverse as signal processing (42), proteomics (43–46), genetics (47), and MRI (48). The main benefit of a fingerprinting approach compared to model-based analysis is that it does not require an a priori assumption of the type of diffusion. Previously developed classification methods train on simulated data and assume the transferability of the results to experimental data. In contrast, diffusional fingerprinting both trains and predicts on experimental data. This allows the fingerprint to agnostically describe a wide range of diffusional systems and diffusional trait classifications using a simple machine-learning classifier. Furthermore, it allows the use of representation learning, offering automatic identification of the representation that best supports the discriminate task at hand. By ranking the predicted features of relevance, the diffusional fingerprint offers mechanistic insights into the differences among the diffusing particles under investigation.

We assessed the ability of diffusional fingerprinting to identify particles in both simulated state-shifting and anomalous diffusion and across multiple diverse experimental systems (e.g., lipases diffusing on native substrates, transcription factors diffusing in cells, or nanoparticles diffusing in mucus on a lipid membrane). We found that diffusional fingerprinting accurately assigned diffusional traits to conditions, allowing for both identification and extraction of key insights, regardless of the underlying diffusion type. By relying on the same 17 features for all classifications, the fingerprint provides a unifying way of mapping a wide range of diffusional phenomena over a common space.

## Results

**Fig. 1.**
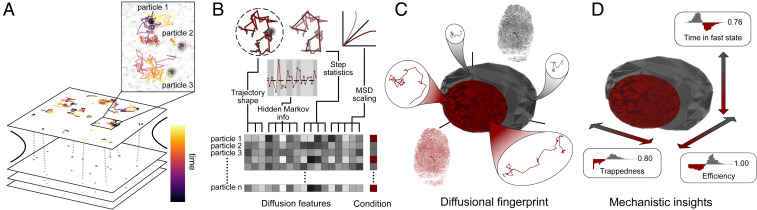
The concept of diffusional fingerprinting for analyzing and classifying molecular identity based on SPT data. (*A*) A typical input consists of SPT data obtained by tracking particles in a recorded movie, visualized here as the horizontal planes in the cartoon. Zoom-in: Three typical trajectories (note the different diffusional behaviors). (*B*) Each trajectory is analyzed and 17 descriptive features underlying SPT diffusional behavior are extracted. The feature values are shown with a gray color code in the horizontal lines of the matrix; these values contain information on the confinement effects, state-shifting diffusion, anomalous diffusion, and non-Brownian displacements. The procedure is repeated for all particle types and conditions, as shown by the color next to the feature matrix. (*C*) The diffusional fingerprint is composed of the combined feature distributions for each particle type, here shown as a dimensionality-reduced plot, where the surfaces encapsulate 1σ of the data points. The diffusional fingerprint of each variant contains information on all observed trajectories. New, unknown trajectories are classified with high accuracy in terms of the known fingerprints, using a simple logistic regression model. (*D*) Ranking of features offers deconvolution of the most relevant differences between fingerprints and gives key mechanistic insights into diffusional differences between measured conditions and particles.

As input to our method, we consider the output generated by a particle tracker: a set of localizations for each particle yielding a dataset of trajectories (Fig. 1*A*). These trajectories can display a variety of diffusion behaviors that the fingerprint needs to capture, including confinement effects, state-shifting diffusion, anomalous diffusion, or non-Brownian displacement.

The fingerprint is based on 17 features chosen to capture most of these phenomena. They consist of 8 features recently proposed in the literature, several features classically used for diffusion analysis, and a set of features based on fitting the displacement trajectories with a four-state hidden Markov model (HMM) with Gaussian emissions. The fitted HMM provides a representation learning platform with improved descriptive power on heterogeneous state-shifting diffusion. The states and transition probabilities are fitted globally across all trajectories. The Viterbi path is computed for each trace, allowing for computation of the residence times in each state (T0, T1, T2, and T3, respectively), along with the average residence time (<tau>). Apart from the HMM features, the feature set includes classical features that are used to describe anomalous diffusion. We fitted a power law to the single-molecule mean-squared displacement (MSD), yielding two estimates of the anomalous diffusion exponent: a diffusion constant and a *P* value (alpha, MSDratio, D, and Pval). To capture the persistence of motion and confinement, we computed four features based on the trajectory shape (kurtosis, dimension, efficiency, and trappedness) and a single feature to capture non-Brownian displacements (Gaussianity). Finally, to describe general trends in the trajectory, we included the average speed, track duration, and MSD value (meanSL, meanMSD, and N). Each of these 17 features’ values is computed for each trajectory. While some of these descriptors have overlapping interpretations, they collectively contribute to a nuanced description of the diffusional process (Fig. 1*B*).

A population of identically diffusing entities will have a diffusional fingerprint with a distribution of features. To compare the distributions, we trained a simple logistic regression classifier to predict the identity (i.e., which experimental condition produced the trajectory) and rank the most relevant features in the prediction by linear discriminant analysis (LDA) projection (Fig. 1*C*). Logistic regression was chosen, as it performed the best across classification tasks while training rapidly and without the need for hyperparameter optimization (*SI Appendix*, Fig. S1). Based on the prediction’s accuracy, one can quickly decide whether two diffusion processes are inherently different and use the ranked features to infer important differences in microscopic motion, thus making it possible to extract key mechanistic information about the systems under investigation.

## Concept Validation: Fingerprinting on Simulated Data

To assess the effectiveness and generality of our fingerprinting method, we initially compared fingerprints on simulated data. Since diffusional differences can manifest themselves in terms of state-shifting diffusion (19), different diffusion rates (49), or particle confinement (50), we chose to evaluate the method’s ability to identify all three. We generated two datasets: one to assess fingerprinting on speed-switching trajectories with different diffusion rates and one to assess fingerprinting on different degrees of confinement.

Two types of speed-switching trajectories were generated using an HMM with four normal diffusion states, 10% transition probability, and two sets of occupation probabilities, yielding both a fast-moving and a slow-moving variant. Three different degrees of confinement were generated by simulating fractional Brownian motion with three different MSD scaling coefficients, giving a subdiffusive variant (α=0.5), a normally diffusive variant (α=1), and a superdiffusive variant (α=1.5). We chose the states, timescales, and number of frames to mimic the experimental systems found in single-molecule cell studies, in which the traces are rather short, states can overlap, and diffusion constants are in the micrometer regime. For each variant, 5,000 trajectories were generated at 0.1-s intervals, where each trajectory was 40 frames long (*SI Appendix*).

**Fig. 2.**
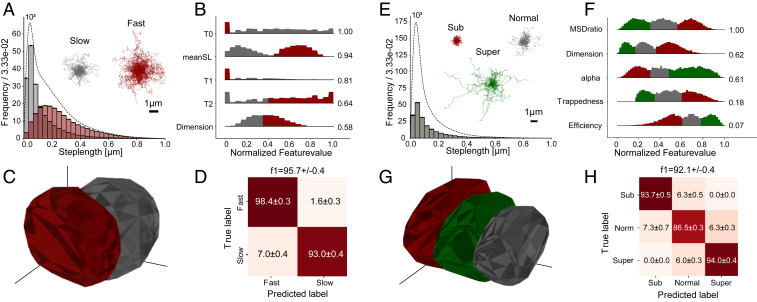
Feature ranking and machine-learning prediction for data were simulated using a four-state diffusion model with two different HMM occupation probabilities (*A–D*) and again using the same HMM occupation probabilities but with three different persistences of motion: HMM diffusion with subdiffusive states (α=0.5), HMM diffusion with normal diffusion states (α=1), and HMM diffusion with superdiffusive states (α=1.5) (*E–H*) (see *SI Appendix* for simulation method). (*A* and *E*) Histogram of step lengths for each variant and distribution for the entire dataset (dotted line). The overlaid trajectories are 100 randomly chosen traces for each variant. The scale bar is the same for both groups of traces (*n* = 10,000 for *A* and *n* = 15,000 for *E*). (*B* and *F*) Normalized distribution of the four most descriptive features in the diffusional fingerprint based on a ranking of components in a one-dimensional LDA projection (numbers to the right). Histogram bins are colored based on the variant with highest value for that bin. (*C* and *G*) Three-dimensional PCA projection of the data with convex hull polygons surrounding 1σ of the data points from the mean for each variant. (*D* and *H*) Confusion matrix for prediction with a logistic regression model trained to separate the fingerprints. The uncertainty is obtained from a stratified fivefold cross-validation with prediction on 20% of the data and training on 80%.

The two speed-switching variants had step-length distributions with a high degree of overlap due to their occupancy in similar HMM states (Fig. 2*A*). Of the 17 descriptive features, we readily identified those with the greatest discernibility to be T0, T1, and T2 (residence times in the three slowest Markov states obtained by fitting an HMM across each trajectory) and meanSL (average step length) from their weights in a one-dimensional (1D) LDA projection (numbers next to histograms in Fig. 2*B*). Interestingly, the fractal dimension, which indicates whether a trajectory is linear, space filling, or confined, also ranked in the top five features, even though all of the HMM states were Brownian and thus should have had an identical fractal dimension equal to 2 (*SI Appendix*, Figs. S2 and S3). However, the mean prediction accuracy was unaltered despite training on only the top four ranking features (*SI Appendix*, Fig. S4), meaning that no other features were required. The key discerning features therefore appear to be readily identified through feature ranking for simulated speed-switching diffusion.

To realize a visual and qualitative representation of the diffusional fingerprint we performed a principal component analysis (PCA), taking the 17 original features and condensing them into three principal components. We plotted the surfaces containing 1σ of the data from the mean (Fig. 2*C*), and the distributions looked surprisingly separable compared to the step-length distributions (Fig. 2*A*). Training a logistic regression classifier on the original 17 features resulted in an F1 score of 95.7 ± 0.4, with classification accuracies of 98.4 ± 0.3% for the fast variant and 93.6 ± 0.4% for the slow variant. We evaluated classification accuracy using the discordance between the ground truth and predicted labels using the confusion matrix, where rows refer to ground truth and columns refer to predicted labels (Fig. 2*D*). A gradient boosted decision tree did not lead to improved accuracy (*SI Appendix*, Fig. S5), suggesting that the optimal separation boundary in this case was linear. Moreover, the relatively high prediction accuracy was a strong indication that the fingerprint allowed accurate classification of particles that were transiently shifting between different speeds.

The step-length distributions for the simulated data with a subdiffusive, a superdiffusive, and a normally diffusive variant were identical for all as they were generated with the same HMM state (Fig. 2*E*). The highest-ranked features in the LDA projection all described trajectory shape or directionality and had discernible multimodal distributions (Fig. 2*F* and *SI Appendix*, Fig. S6). Even though the variants had identical diffusion states, their trajectories could be classified based on trajectory shape, with an F1 score of 91.1 ± 0.4% and respective accuracies of 93.7 ± 0.5% for the subdiffusive variant, 94.0 ± 0.4% for the superdiffusive variant, and 86.5 ± 0.3% for the normally diffusive variant (Fig. 2*H*). The slightly lower prediction accuracy for the normally diffusive variant most likely originated from normal diffusion lying between sub- and superdiffusion in its highest-ranked features, leading to error for traces whose features overlapped between the three variants. This error was removed by increasing the trajectory duration and resulted in similar classification accuracies across all variants (*SI Appendix*, Fig. S7). Thus, diffusional fingerprinting accurately classified simulated particles exhibiting different, yet overlapping speeds with varying degrees of confinement, while correctly identifying the relevant features for prediction.

Finally, we generated a stress test to further support performance improvement and benchmark the approach against algorithms currently employed in diffusion classification. Since diffusional fingerprinting employs a different classification setting than do the current methods, a direct comparison is generally not possible. However, in the special case of simulated datasets, where the two classification settings are identical, such a comparison can be made. We compared diffusional fingerprinting against a feature-based classifier containing no HMM-based features and against a convolutional neural network (CNN) previously employed for diffusion classification (32). A benchmark was made for both high and low localization errors and for short traces, using the traces obtained from low signal-to-noise movies with high particle densities. Across all cases, we found that our features outperformed the currently used feature sets (F1 scores of 88% vs. 82%, 76% vs. 67%, and 79% vs. 66%) and performed on par with a state-of-the-art CNN (on par for low localization error data, slightly better for high localization error, and slightly worse for high background with short traces; *SI Appendix*, Table S2 and Figs. S8–S10), while at the same time outputting feature rankings for mechanistic insights.

## Fingerprinting Allows for Precise Identification of Enzymes with Identical Catalytic Efficiency

**Fig. 3.**
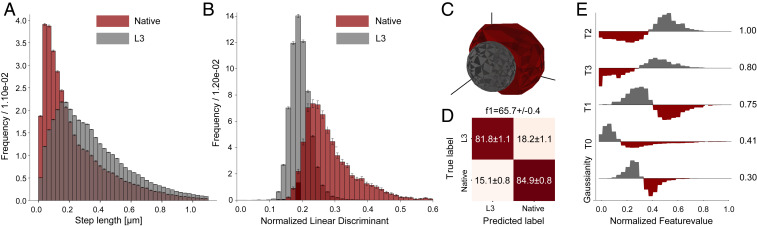
Diffusional fingerprinting of SPT data for two functionally similar TLL variants, L3 and native (19). (*A*) Distribution of steps between frames of 100 ms for tracked particles of the two variants (*n* = 3,016,313). (*B*) One-dimensional LDA projection of features for the two variants (*n* = 68,200 for L3 and *n* = 5,630 for native). (*C*) Three-dimensional PCA projection of diffusional fingerprint features. Spheres represent the convex hull containing 1σ of the data from each group’s mean. (*D*) Confusion matrix for a stratified fivefold cross-validation with 20% validation and 80% training data for a logistic regression classifier. Uncertainties denote standard deviation across the five cross-validation runs. The F1 score is lower than the accuracies, due to the strong data imbalance between the two classes (68,200 L3 and 5,630 native). (*E*) Differential histograms of the five features with the highest LDA projection matrix components used to make *B*. Histograms are colored based on the variant with the highest value in each bin; feature values have been normalized for visual comparison. Numbers denote the normalized vector component in the LDA projection (*n* = 73,830).

To evaluate the utility of fingerprinting on real data, we initially analyzed a subset of our published SPT data on two fluorescently labeled variants of *Thermomyces lanuginosus* lipase (TLL) (19). TLL is an interfacially activated hydrolase that contains an amphipathic helix known to tightly regulate its function. Here we investigated two TLL variants, native TLL and L3. The L3 variant differs from the native TLL by having a lid region that is mixed between TLL and *Aspergillus niger* ferulic acid esterase (FAEA) (51). The native and L3 lipases display practically identical catalysis rates in uninhibited conditions (19, 51, 52), and given that enzymatic turnovers have been found to correlate to diffusion (49, 53–55), one might expect a similar diffusion for variants of similar catalytic efficiency and size. This is confirmed by the two variants’ significant overlap in step-length distribution (Fig. 3*A*). The native variant, in general, displays slower diffusion, but distinguishing the two variants by step-length distribution alone would be challenging.

Computation of the diffusional fingerprint and LDA projection resulted in two quite separable distributions (Fig. 3 *B* and *C*). Using a logistic regression and stratified fivefold cross-validation, we predicted L3 with an accuracy of 81.8 ± 1.1% and native with an accuracy of 84.9 ± 0.8%, for an F1 score of 65.7 ± 0.4% (the score was lower than the accuracies due to label imbalance) (Fig. 3*D*). The diffusional fingerprint was thus found to deconvolute the two overlapping, and otherwise indiscernible, step-length distributions. Furthermore, fingerprinting allowed us to decipher the underlying differences behind the distinctly different motions. The highest-ranked features were the residence times in each of the HMM diffusion states, with L3 spending more time in the faster states (T2 and T3) and native spending more time in the slower states (T0 and T1) (Fig. 3*E* and *SI Appendix*, Fig. S11). Such difference indicates that L3 displays longer sequences of larger jumps during a trajectory compared to the native variant.

The differences brought forth by this analysis show that L3 and native’s similar catalysis is realized by means of different diffusional strategies: L3 stochastically makes large jumps, whereas native diffuses more unimodally. This suggests that L3 turnovers fuel leaps that allow the enzyme to step away from product regions, indicating antichemotaxis (56). An antichemotactic behavior could make L3 less prone to product inhibition compared with native TLL; indeed, L3 has been found to display limited bulk product inhibition (19). This mechanistic conclusion was reached in a fast, agnostic, and intuitive manner, highlighting the strength of the diffusional fingerprint.

Logistic regression is a rather simple classifier, and we therefore tested whether further improvement could be made to its prediction accuracy by applying a more complex model. To investigate this, we tested two neural network architectures: a CNN previously proposed for diffusion classification (32) and a long short-term memory (LSTM) bidirectional neural network (a method previously employed by our laboratory for classifying fluorescence resonance energy transfer [FRET] time series) (57). The LSTM was trained to classify the variants based on their raw step lengths and position data, and the CNN was trained on raw positions. The LSTM and CNN classifiers led to F1 scores of 97 and 95% for LSTM and CNN, respectively. The L3 variant was classified with an accuracy of 93 and 95% for LSTM and CNN, respectively, and the native variant was classified with an accuracy of 89 and 87% for LSTM and CNN, respectively (*SI Appendix*, Fig. S12). A slight increase in prediction accuracy is expected, as the set of 17 features might never be completely optimal. Further improvement on the network architecture may result in increased accuracy relative to the fingerprint, but this extends beyond the scope of the current work. However, since the modern neural network led only to marginal improvement in classification accuracy, at the expense of mechanistic insights, the descriptive power inherent in the few chosen features of the fingerprint is surprisingly strong.

## Universal Application of Fingerprinting on Multiple Diverse Systems

Finally, we tested whether diffusional fingerprinting generalizes across systems by evaluating its efficiency on three significantly different biomolecular entities and conditions: L3 lipase diffusing on two different substrates, transcription factors diffusing on DNA in live cells, and differently coated nanoparticles diffusing in mucus on a lipid membrane.

**Fig. 4.**
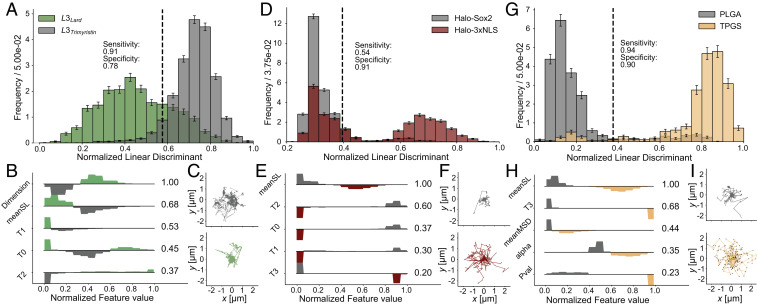
Diffusional fingerprinting applied to three widely diverse systems. *A*, *D*, and *G* display the one-dimensional LDA projection showing a threshold that maximizes the sum of sensitivity (proportion of correctly identified positives) and specificity (proportion of correctly identified negatives). *B*, *E*, and *H* display the features ranked by their components in the LDA projection. *C*, *F*, and *I* display 30 exemplary traces from the respective dataset used to construct *B*, *E*, and *I*. (*A–C*) Diffusional fingerprinting of the TLL L3 variant measured on two different substrates, lard and trimyristin (*n* = 6,270). (*D–F*) Diffusional fingerprinting of the two halo-tagged transcription factors Sox2 in mouse embryonic stem cells and NLS in human U2OS cells from a study by Hansen et al. (58) (*n* = 8,937). (*G–I*) Diffusional fingerprinting of differently coated nanoparticles diffusing in mucus on top of a lipid membrane (*n* = 2,286). The confusion matrix from a fivefold stratified cross-validation of a logistic regression classifier for each dataset is shown in *SI Appendix*, Fig. S13.

The first dataset consists of the TLL variant L3 measured on two distinctly different substrates, trimyristin and lard (*SI Appendix*). The diffusional fingerprint allowed for an F1 score of 85.1 ± 0.7% and classification accuracies of 90 ± 1% for predicting the trimyristin surface and 78 ± 3% for predicting the lard surface (Fig. 4*A* and *SI Appendix*, Fig. S13*A*). It appears, as expected, that interchanging the substrate leads to significant changes in lipase movement. The most important feature was the fractal dimension, with a higher fractal dimension for diffusing on lard than on trimyristin (Fig. 4*B*). The other important features (meanSL, T1, T0, and T2) originated from L3 diffusing faster on trimyristin than on lard (*SI Appendix*, Fig. S14). This result may be interpreted as lard forcing a slower and more confined diffusion on the enzyme than trimyristin, an effect that is also evident in the raw trajectories (Fig. 4*C*). The fact that this prediction was based on features different from those separating the L3 and native TLL variants highlights the generality of the fingerprinting method.

We next tested our method on published results for transcription factors diffusing in cells (58, 59). Here, the transition between bound and free diffusion led to different trajectories, depending on the affinity for the DNA and the free diffusion constant. Hansen et al. (58) characterized the trajectories using a two-state model with both a bound state and a freely diffusing state. We looked at two of the four variants studied by Hansen et al. (58): Sox2, a largely freely diffusing protein found in mouse embryonic stem cells that has an intermediate diffusion constant, and nuclear localization signal (NLS) in human U2OS cells, which has a lower binding affinity to DNA than does Sox2 and displays a faster diffusion constant. Using the fingerprinting methodology, we attained an F1 score of 72.7 ± 0.1% and predicted NLS motion with 91 ± 2% accuracy and Sox2 motion with 52 ± 2% accuracy (*SI Appendix*, Fig. S13*B*).

Inspecting the projected features revealed that the asymmetry between classification accuracies was due to the existence of two distinct populations within the NLS feature distributions (Fig. 4*D*). This overlap can be visualized as the appearance of fast traces for NLS that were not seen for Sox2 (Fig. 4*F*). Training with a bidirectional LSTM or a CNN on the step length and coordinates showed no further improvement, suggesting that this overlap is inherent in the diffusional dynamics of the system and that fingerprinting precisely resolved the difference (*SI Appendix*, Fig. S15). The most relevant feature for prediction was the average step length (meanSL), and Hansen et al. (58) found similar diffusion constants to those obtained from the meanSL histogram (12 μm/s for the NLS peak and 3$\mu m^{2}/s$ for Sox2), confirming that diffusional fingerprinting readily identifies the key discerning diffusional properties for identification (Fig. 4*E* and *SI Appendix*, Fig. S16).

Finally, we tested the ability of diffusional fingerprinting to accurately predict and annotate particles in a completely different system: nanoparticles with two different types of coatings diffusing in mucus on top of a lipid membrane. The effect of polymer coating to increase mucus permeability was investigated by directly comparing the mobility of pure polylactic-co-glycolic acid (PLGA) nanoparticles to specially designed mucus inert particles with an enzymatically cleavable shell of d-α-tocopheryl polyethylene glycol 1000 succinate (TPGS) (12). Here, the universality of diffusional fingerprinting was manifested by its ability to classify TPGS particles with a prediction accuracy of 93 ± 1% and PLGA particles with an accuracy of 91 ± 1% (Fig. 4*G* and *SI Appendix*, Fig. S13*C*).

Feature ranking revealed the high prediction accuracy to be due to the mechanistic origin of varying diffusion speeds and differing confinement. Particles coated with TPGS displayed a greater average step length than did raw PLGA particles and had a lower occupancy in the fastest Markov state. Not only were the TPGS particles faster than the raw particles, but also their motion was less confined and more Brownian, as seen by the increased alpha centered around 1, a higher meanMSD, and an estimated fractal dimension close to 2 (Fig. 4 *H* and *I* and *SI Appendix*, Fig. S17). Since subdiffusion can be related to diffusion on a fractal (60), it is possible that the PLGA particle was constrained by mucus interactions to move on a lower-dimensional manifold defined by channels in the mucus and that this constraint was lifted by the TPGS coating, allowing the particle to diffuse in a Brownian fashion. While previous analyses of these trajectories also identified the increase in diffusion speed (12), the observation that TPGS coating acts by lifting a subdiffusive-like state is completely different.

## Discussion

Here we introduced the concept of diffusional fingerprinting, an approach that enables classifying and describing SPT trajectories regardless of underlying diffusion type. We demonstrated how a trained classifier can be used to predict variants and how 1D LDA projection allows for precise outputting of the diffusional traits that sets variants apart. By relying on the same 17 features for each classification, single-particle diffusional fingerprinting provides a unified way to map a wide range of diffusional phenomena to a common space.

Since the fingerprint is a distribution of features, the degree of overlap in feature values decides the separability of diffusional fingerprints. The simulated data were deliberately chosen to be short (40 frames per trajectory) to faithfully represent challenging SPT in cells (Fig. 2). Increased imaging time greatly suppresses error, and we found accordingly that the increased imaging time and decreased localization error greatly improved the separability of the fingerprinting distributions, and thus the classification accuracy, across all simulated datasets (*SI Appendix*, Figs. S7, S9, and S10). While most of the feature distributions may be explained from this, it is possible that part of the spread in the fingerprint originates from a suboptimal selection of features. This was suggested from the observation that a bidirectional LSTM neural network and a CNN slightly improved the fingerprinting prediction accuracy on the native and L3 datasets (*SI Appendix*, Fig. S12). Features can always be improved, and as better features and classifiers are employed in the future, our implementation of diffusional fingerprinting may be further extended. However, no improvement was found when training on the transcription factor dataset (*SI Appendix*, Fig. S15) and the method performed on par with a CNN on the stress test dataset (*SI Appendix*, Table S2). These comparisons suggest that while slight improvements to the features might be possible, most of the relevant information is captured in the 17 chosen features.

We have focused on the case of labeled data in our investigation, but the descriptive power of the features used for the diffusional fingerprint naturally extends to unlabeled data, as the features do not need a label for computation. Dimensionality reduction and clustering techniques could be used to identify traces in a dataset with distinct diffusion characteristics and extract their diffusional fingerprints in an unsupervised fashion from fingerprinting clusters. The fact that the diffusional fingerprint precisely outputs features in a common space across a range of diverse biomolecular systems strongly supports that the proof-of-principle uses of diffusional fingerprinting outlined here are only a few of the many possibilities for this technique. We envision its application across systems or laboratories, generating libraries of conditions for diverse types of motion. The optimal number of HMM states could be iteratively updated using a variational approach as more data are added (61). Once a dictionary of diffusional traits is generated, a pattern recognition algorithm based on machine learning may output the feature classification and output identity. This may be type of particle, biomolecular recognition, ligand-mediated conformational change, topographical or geometrical variation in the diffusion medium from high-throughput screening analysis, or a theoretical diffusion model, as well as different mechanisms or pathways for the cellular entry of viruses or nanocarriers.

## Supplementary Material

Supplementary File

## Data Availability

All tracked data used for fingerprint generation and single-particle tracking data have been deposited in ERDA, University of Copenhagen (https://sid.erda.dk/cgi-sid/ls.py?share_id=ctvcZhDnE7) (62). Previously published data were used for this work [Wan et al. (12), Bohr et al. (19), and Hansen et al. (58)]. Code for computing the fingerprints and plotting feature projections is available on GitHub (63).
