# Supplementary material for: Single-particle diffusional fingerprinting: A machine-learning framework for quantitative analysis of heterogeneous diffusion
Source: Proc Natl Acad Sci U S A. 2021 Jul 28;118(31):e2104624118. doi: 10.1073/pnas.2104624118 (PMC8346862; doi:10.1073/pnas.2104624118)
Supplement: Supplementary File [file pnas.2104624118.sapp.pdf]

PNAS
www.pnas.org

Supplementary Information for

Single Particle Diffusional Fingerprinting: A machine learning framework for quantitative analysis of heterogeneous diffusion

Henrik Pinholt, Søren S.-R. Bohr, Josephine Iversen, Wouter Boomsma and Nikos S. Hatzakis

Nikos S. Hatzakis. E-mail: hatzakis@nano.ku.dk

This PDF file includes:

Supplementary text
Figs. S1 to S17 (not allowed for Brief Reports)
Tables S1 to S2 (not allowed for Brief Reports)
SI References

Supporting Information Text

Simulation of diffusion traces

Normal diffusion with four HMM states. The diffusion with normal persistence and varying step lengths was generated stepwise starting from (x,y)=(0,0) with a diffusion constant drawn from: ($0.006\,\mu m^2/s$, $0.025\,\mu m^2/s$, $0.1\,\mu m^2/s$, $0.4\,\mu m^2/s$) with probabilities (0.4, 0.25, 0.25, 0.1) for slow and (0, 0, 0.5, 0.5) for fast. The next diffusion constant is redrawn with probability 0.1 and kept the same with probability 0.9. This algorithm leads to a transition probability matrix of

$$A = \begin{pmatrix} 0.9 + 0.1p_1 & 0.1p_1 & 0.1p_1 & 0.1p_1 \\ 0.1p_2 & 0.9 + 0.1p_2 & 0.1p_2 & 0.1p_2 \\ 0.1p_3 & 0.1p_3 & 0.9 + 0.1p_3 & 0.1p_3 \\ 0.1p_4 & 0.1p_4 & 0.1p_4 & 0.9 + 0.1p_4 \end{pmatrix} . \qquad [1]$$

For each frame, a displacement is drawn from the normed displacement distribution of a 2D random walk

$$P(r) = \frac{r}{2D_i t} e^{-\frac{r^2}{4D_i t}} , \qquad [2]$$

where D_i is the diffusion constant of the current state i and t=0.1 s is the frame length (the temporal resolution used for the the L3-Native lipase data set). A random direction is chosen and the displacement is added to the current coordinates. All traces generated were 40 frames long and 5000 trajectories were generated for each of the 5 variants.

Super/Sub diffusion with four HMM states. The parameters for the HMM state are identical to the slow variant: (0.4, 0.25, 0.25, 0.1) with the same transition probability of 0.1. To simulate increments with statistics of a given α, the fbm python library with the daviesharte method was employed. For a given α, traces of unit diffusion constant were generated from a series of fbm increments with the `fgn` method of the library. For each increment in the generated trace, the increment was scaled with $\sqrt{2D_i\Delta t}$ where D_i is the diffusion constant for the state at that frame and Δt was set to 0.1 ms as in the simulation for normal diffusion.

Computation of the features

In order to provide a generalizable yet informative description of single particle tracking data, we chose to use feature-based machine learning for the prediction of mutant variants. The workflow is presented in Fig. 1, and consists of the following steps: Given a set of trajectories (x, y), we compute a set of 17 intuitive features aimed to describe general trends of random walks in many environments of interest (see table S1). The features are described in the following. The features Trappedness, kurtosis, gaussianity, fractal dimension, efficiency, and MSDratio are adapted from Kowalek *et al* (1).

Table S1. Features used for the single molecule diffusional fingerprint as well as a short description of what they seek to describe. The features T0, T1, T2, T3, <tau>, and meanSL are obtained from the step lengths of the trace and give information on how fluctuating the diffusion speed is over the trajectory and the average speed of the diffusion. The features Pval, alpha, D, trappedness, meanMSD, and MSDratio gives information on how much the particle spreads out during the trajectory. The features kurtosis, gaussianity, fractal dimension, and efficiency allow the fingerprint to capture directionality of the walk by describing correlations between steps as well as linearity and asymmetry of the trajectory. Finally, the duration of the state was included as it gives key information on binding affinity and bleaching.

Feature name	Description
T0	Time in the slowest diffusion state
T1	Time in the second slowest diffusion state
T2	Time in the second fastest diffusion state
T3	Time in the fastest diffusion state
<tau>	Average residence time in a diffusion state
meanSL	Average step length for the trace
Pval	Quality of a power law fit to the msd curve
alpha	MSD power law scaling coefficient (<1 for subdiffusive and >1 for superdiffusive)
D	Diffusion constant from power law fit
Trappedness	Estimates whether the walker is trapped ($\approx$ 0.5 for Brownian motion)
meanMSD	Intermediate time spread of the trajectory
Kurtosis	Heaviness of the tails in the distribution of points in the entire trajectory ($\approx$ 2 for Brownian motion)
Gaussianity	Heaviness of the tails of the distribution for the steps in the trajectory (1 for Brownian motion)
Fractal dimension	Space-filling-ness of the trajectory (slightly less than 2 for Brownian motion)
Efficiency	Linearity of the trajectory (-7 for Brownian motion)
MSD ratio	MSD power law scaling coefficient (from statistics of trajectory rather than fit, 0 for Brownian motion)
N (track duration)	Bleaching rate or binding affinity to the substrate

A. Features based on HMM fitting: T0, T1, T2, T3 and <tau> . A four state Hidden Markov model with Gaussian emission distributions is fitted to the step lengths of all traces. The fitting of the distribution and transition matrix is done using the function `HiddenMarkovModel.from_samples()` from the pomegranate library in python with `n_components = 4` and the distributions using the pomegranate `NormalDistribution` class for distributions. With the trained model, the Viterbi path is computed for each trajectory, and the features T0, T1, T2, and T3 are computed as the fraction of frames spent in each of the four states along the trajectory. Finally, the feature <tau> is computed as the average residence time in states along the trajectory. We chose Gaussians and not the distributions of step length for normal diffusion since the purpose of the HMM is not to fit the actual diffusion model, instead it is meant to capture the rough step length regimes in the data, and for this, the normal distribution is nicely suited as it is more localized than the broader step length distributions of Brownian motion.

Both for motion of lower and higher complexity than four states, the chosen value of four HMM features was found to lead to good performance. The transcription factor data used for Fig. 4 D, E, F, which was previously analyzed using a two-state model (2), could be analyzed in terms of our four states to retrieve similar mechanistic interpretations while maintaining a classification score on par with deep neural network classifiers (Fig. S8). The comparably simple four-state HMM features performed well on a complex data set consisting of several state-shifting species based on both normal diffusion, directed motion, confined diffusion and anomalous diffusion. The diffusional fingerprinting feature set yielded a significant improvement in classification as compared to non-HMM features and the current state-of-the-art in convolutional neural network (CNN) classifiers (Table S2, Fig. S11, S12 and S13).

B. MSD fitting based features: Pval, alpha and D . We compute the mean squared displacement for each trace and fit the resulting curve to a power law: $\mathrm{MSD} = Dt^{\alpha}$. The diffusion constant D gives information on the general spreading of the motion, whereas α is a measure of persistence in the walk. $\alpha = 1$ corresponds to normal diffusion, whereas $\alpha > 1$ is superdiffusive and persistent while $\alpha < 1$ yields subdiffusive motion that is anti-persistent and often trapped (3). Finally, we include the p-value in a chi2 test for the fit as a feature since it will give a rough estimate as to what degree the model fits with a power law curve.

C. Trappedness. The trappedness is based on a computation done by Saxton for the probability P that a normal random walk with diffusion constant D (in units length^2/frame) will stay within a circular region of radius R for a time t (4)

$$P = \exp\left(0.2045 - 0.25117\left[\frac{Dt}{R}\right]\right) . \qquad [3]$$

If this probability is deviating significantly from 1, one would start to think that the particle might be trapped. Therefore, the feature computed as $1 - P$ becomes a measure of the trappedness of the particle

$$\mathrm{Trappedness} = 1 - P = 1 - \exp\left(0.2045 - 0.25117\left[\frac{Dt}{R}\right]\right) . \qquad [4]$$

The diffusion constant is estimated from the difference between the first two points in the MSD curve, and R is taken to be the half the maximum separation between any two points in the trajectory. From simulations we found that the trappedness is distributed centrally around 0.5 for normal diffusion.

D. Kurtosis. Kurtosis is a feature which compares the tail of the spatial probability density of the trajectory to that of the Gaussian (1). For a given trajectory of duration N with coordinates $(x_i, y_i), i = 1, 2, .., N$ this is done by first computing the covariance matrix (or gyration tensor)

$$\boldsymbol{T} = \begin{pmatrix} \frac{1}{N}\sum_{j=1}^{N}(x_j - \langle x\rangle)^2 & \frac{1}{N}\sum_{j=1}^{N}(x_j - \langle x\rangle)(y_j - \langle y\rangle) \\ \frac{1}{N}\sum_{j=1}^{N}(x_j - \langle x\rangle)(y_j - \langle y\rangle) & \frac{1}{N}\sum_{j=1}^{N}(y_j - \langle y\rangle)^2 \end{pmatrix} . \qquad [5]$$

For normal diffusion, the eigenvectors of this matrix will point in the directions of the minor and major axis of an ellipse enclosing values of equal probability. The major axis of this ellipse will be in the direction of the eigenvector (r_1, r_2) with the largest eigenvalue. Given the major axis of the covariance matrix, we can project onto this axis to get a set of scalar values for each coordinate (x_i, y_i) yielding a maximum spread 1D projection x_i^p of the trajectory

$$x_i^p = r_1 x_i + r_2 y_i . \qquad [6]$$

It is the kurtosis of the distribution for x_i^p to which the feature name refers. Kurtosis is the fourth moment of a distribution and it is estimated for the maximum spread projection as

$$\kappa = \frac{1}{N}\sum_{j=1}^{N}\frac{(x_j^p - \langle x\rangle)^4}{\sigma_{x^p}^4} , \qquad [7]$$

where σ_{x^p} is the estimated standard deviation of the projected trajectory. The kurtosis captures how heavy-tailed the distribution is. As a feature, it gives a comparison of how far out the edge-point of a trajectory gets compared to what would be expected for a normal Brownian motion. Simulations show that normal diffusion displays a kurtosis feature value around 2. Therefore, a kurtosis larger than 2 suggests that the motion is superdiffusive, and a kurtosis less than 2 suggests subdiffusive motion. However, since one needs to both estimate the mean and variance before being able to compute the kurtosis, for very short trajectories, the estimation error will be quite large, which is probably why it is not the most descriptive feature for the relatively short trajectories analyzed in single molecule studies where trajectories rarely get much longer than a 100 frames.

E. Gaussianity. Whereas kurtosis describes the spatial distribution of the whole trajectory, gaussianity seeks to investigate the spatial distribution at the time-scale of single frames (5). The gaussianity is based on the ratio between the quartic moments

$$\langle r_n^4 \rangle = \frac{1}{N-n} \sum_{i=1}^{N-n} (x_{i+n} - x_i)^4 + (y_{i+n} - y_i)^4 \,, \qquad [8]$$

and the second moments

$$\langle r_n^2 \rangle = \frac{1}{N-n} \sum_{i=1}^{N-n} (x_{i+n} - x_i)^2 + (y_{i+n} - y_i)^2 \,, \qquad [9]$$

of the trajectory. The ratio of these two gives a set of numbers for each frame n. For a Brownian diffusion in 2D, the ratio becomes 2, and therefore we divide by 2 such that the gaussianity becomes 1 for Brownian motion. Values larger than 1 means that the step distribution is narrower than a those for Brownain motion, and values smaller than 1 means that it is wider.

$$g_n = \frac{\langle r_n^4 \rangle}{2 \langle r_n^2 \rangle^2} \,. \qquad [10]$$

Since the gaussianity is defined for each n as an average over increments n frames apart, we have N such gaussianities corresponding to N different timescales of the diffusion. We chose to use the average gaussianity along the trajectory as our feature, and only compute up to $n = N/2$ separations as the number of data points for computation of the moments becomes very sparse as you increase n and thus yielding a quite large variance. The resulting formula for the gaussianity features is

$$G = \frac{2}{N} \sum_{i=1}^{N/2} g_i \qquad [11]$$

F. Fractal dimension. The fractal dimension is a measure of how space filling the points of the trajectory is. Using the estimator by Katz and George (6), we estimate it as

$$D_f = \frac{\ln(N)}{\ln(NdL^{-1})} \,, \qquad [12]$$

where L is the total length of the path, N is the number of steps in the trajectory and d is the largest distance between any two points in the trajectory. The fractal dimension is around 1 for straight trajectories (ie. ballistic motion), around 2 for normal diffusion and larger than 2 for subdiffusion (ie. confinement). For normal diffusion the distribution has been previously found to be consistent with a lognormal and peaked slightly below 2 (6), which we also found in our simulations (see Fig. S14A). We find that as traces become longer, the peak approaches 2 which is the fractal dimension expected for Brownian motion (Fig. S14B). We also found that HMMs consisting of purely normal diffusion states with identical diffusion constants but differing equilibrium occupations display slightly different estimated fractal dimensions (Fig. S14C, Fig. S15). This makes sense as the Brownian emissions are weighted differently on average, leading to an altered expectation for the ratio d/L.

G. Efficiency. The feature efficiency is the ratio of end-to-end distance to the sum of distance traveled for the particle (1)

$$\gamma = \sqrt{\frac{(x_{N-1} - x_0)^2 + (y_{N-1} - y_0)^2}{(N-1)\left[\sum_{i=1}^{N-1} (x_i - x_{i-1})^2 + (y_i - y_{i-1})^2\right]}} \,. \qquad [13]$$

This ratio has the same flavour as the fractal dimension. It will yield similar information, but it is very good at picking up directed motion. We found the distribution of γ in 2D Brownian motion to be roughly lognormal in simulations, with a mean of the $\log(\gamma)$ around -7. Since the distribution is roughly lognormal, and efficiency ratios are rather low, we chose to use the logarithm of the efficiency as the feature in our fingerprint:

$$\mathrm{E} = \log(\gamma) \,. \qquad [14]$$

H. MSD ratio. The mean squared displacement ratio is similar to the fit of the MSD curve. It gives a measure of the shape of the MSD curve, and is defined as (1):

$$\kappa(n_1, n_2) = \frac{\langle r_{n_1}^2 \rangle}{\langle r_{n_2}^2 \rangle} - \frac{n_1}{n_2} \,, \qquad [15]$$

with $n_1 < n_2$. Since the MSD curve often follows a power-law, the MSD-ratio is an estimate of α in $\mathrm{MSD} = Dn^\alpha$ for a set of two point along the MSD curve with the $\alpha = 1$ (normal diffusion) result n_1/n_2 subtracted off. The MSD ratio will be 0 for normal diffusion greater than 0 for superdiffusion and less than 0 for subdiffusion.

I. Other features: meanSL, meanMSD and N. On top of the features described above, we include the average step length and average MSD as a feature. These are meant to provide a general overview of the tendencies in the trace. The HMM residence times: T0, T1, T2 and T3 would only contain equivalent information to the average step length in the limit of an infinite trace. For this reason, the average step length is a rather good feature for describing short traces. The average MSD similarly captures the general spreading of the trace, and is meant to supplement the other features derived from the MSD that might be blurred for short traces. Finally, the duration of the trace is included as a feature since in some systems, like surface based tracking studies with TIRF, the trace duration gives information on binding kinetics in the system.

J. Machine learning methodology. The machine learning approach adopted to separate trace data starts by generating the 17 fingerprint statistics described above and in table S1. This generates a 2D data set $\boldsymbol{X}$ of shape $(N, 17)$ where N is the number of trajectories in the data set. The traces are then labeled giving a vector $\vec{y}$ of shape $(N, 1)$. The data set $\boldsymbol{X}$ is then normalized to zero column mean and unit column variance. Prediction is done using a Logistic Regression classifier which is trained using scikit-learn's function `LogisticRegression()` with `max_iter` set to 10000 and a trainable offset.

The Logistic regression classifier was chosen after a comparison with 7 other classifiers (Fig. S1). Logistic regression was found to perform similar to other classifiers in binary classification and best—but comparable to SVM-based classifiers and a gradient boosted decision tree—in multiclass classification. Logistic Regression was chosen as it has no need for hyper-parameter optimization like SVMs and trains faster than a gradient-boosted decision tree.

In binary classifications, a histogram of the class probabilities estimated with the Logistic Regresssion classifier is plotted for each class, and then a ROC curve is computed for a decision boundary at each histogram bin. The boundary which maximize the sum of sensitivity and specificity on the training data is chosen and used for prediction.

In order to assess the generalizability of the model, a 5-fold cross validation is performed with 20% of the data set aside for testing in a stratified fashion, keeping the fractions of class labels the same in the training and test set. When an overview of the feature distributions is required, the Linear Discriminant 1D projection is computed using the scikit-learn function: `sklearn.discriminant_analysis.LinearDiscriminantAnalysis`.

1. Classification benchmarking

A. Data set simulation. To quantify noise-robustness of the methodology and compare against current state-of-the-art classifiers, we generated three stress-test data sets. The simulation consisted of one normal diffusion variant and three binary HMM variants shifting between normal diffusion and one of either directed motion, confined diffusion or anomalous diffusion. The HMM model employed is visualized in Fig. S11A and we chose p_{shift}=0.1. For each trace, a state history was generated using the HMM and the motion types in each state was generated as described elsewhere (1), with the simulation parameters: Δt=1/30 s, D = 9.02 µm^2/s, α=0.3, R=9, B=4. All data sets consisted of 5,000 traces for each variant giving a total of 20,000 traces out of which 70% was used for training and 30% was used for validation.

The first data set investigates how the models perform on good data. Each trace in this data was chosen to be 100 frames with a low localization error ratio of $Q = \sqrt{4D\Delta t}/\sigma$ =5, for all traces where σ is the standard deviation of the localization error (Fig. S11). The second data allow quantification of the effects of localization errors on classification accuracy. All traces are 100 frames long but five simulations where done with varying degrees of localization error ratios of Q =16, 4, 1, 0.25 and 0.0625 (Fig. S12). The last data sets allow quantification of the effects of short traces often found in high density tracking or low signal to noise movies. Q was fixed at 5 and 7 simulations were done with trace lengths of 20, 40, 80, 160, 320, 640 and 1280 (Fig. S13).

B. Models for comparison. We compare against two classifiers recently proposed in the literature (1). The first is the feature-based classifier proposed by Kowalek *et al.* which contains 8 of our 17 features for classification trained with a Gradient Boosted Decision Tree from the `sklearn` python library with parameters `n_estimators`=500, `max_depth`=10, `min_samples_split`=5 and `min_samples_leaf`=4. The overlapping features are D, MSDratio, efficiency, α, trappedness, kurtosis, fractal dimension and gaussianity. The second classifier is a CNN model proposed in (1) generated by the python module `mcfly` using the method `generate_CNN_model` with filter sizes of 49, 36, 18, 83, 90, 27, 1550 hidden nodes, a learning rate of 0.00021795428728036654, and a regularization rate of 0.0014064205292043147. For easy comparison, we employed the same gradient boosting classifier and parameters as employed for the Kowalek features when training on the fingerprinting features.

2. Acquisition of L3 diffusing on Lard vs. trimyristin

The methodology for surface preparation, data acquisition and analysis is described elsewhere (7). Briefly here, the lipid substrate surfaces were prepared on round 0.25 mm microscope cover glass slides by spin coating 1-2 drops of 14 µL LARD solution (24.5 g/L LARD with 0.05 ppm DOPE-ATTO488 dissolved in toluene) or 12 µL trimyristin-EnzChek solution (20 g/L trimyristin mixed with 0.1 ppm EnzChek505 dissolved in toluene) at 5000 rpm for 2x60 seconds with 10 s break in-between. The glass slides were placed in metal chambers and stored in vacuum for at least 2 hours or overnight before immediate use. Movies were acquired using a Total Internal Reflection Fluorescence (TIRF) microscope (IX 83, Olympus). Videos of 2000 frames and pixel width of 160 nm, were recorded using an EMCCD camera (ImageEM X2, Hamamatsu) and an oil immersion 100x objective (UAPON 100XOTIRF, Olympus). Imaging of the TLL enzyme mutant L3 labelled with Setau647 was performed with 300 EM gain, 80ms exposure time and 101-114 nm penetration depth, excited by 640 nm laser line. Videos were recorded

Table S2. Comparison of diffusional fingerprinting to other classifiers across several simulated datasets. Low localization error data set: 20000 traces 30% validation 70% training consisting of 5000 normal diffusion traces and 5000 traces for each of three Hidden Markov Models (HMMs) converting between normal diffusion and either directed, confined or anomalous diffusion. Simulations were done as described elsewhere (1) with similar parameters (N=100, Δt = 1 / 30 s, D = 9.02 µm^2/s, α = 0.3, N = 100, R = 9, B = 4, Q=5). The HMM model used is shown in Fig. S11 and p_{shift} was set to 0.1. High localization error data set: Same as for the stress-test dataset but Q=1. High background noise: Same as for stress-test data set but N=40.

Dataset	Previous feature based (Interpretable features)	CNN (Non-Interpretable features)	Diffusional fingerprinting (Interpretable features)
Low localization error	82%	88%	88%
High localization error	67%	74%	76%
High background noise or density (Short traces)	66%	74%	79%

in 60 µL 50 mM TRIS buffer pH 9 adding 1-2 µL enzyme solution which gives a lipase concentration of 0.2-0.4 nM in chamber. Two surfaces of both LARD and trimyristin have been used, recording two videos from each surface.

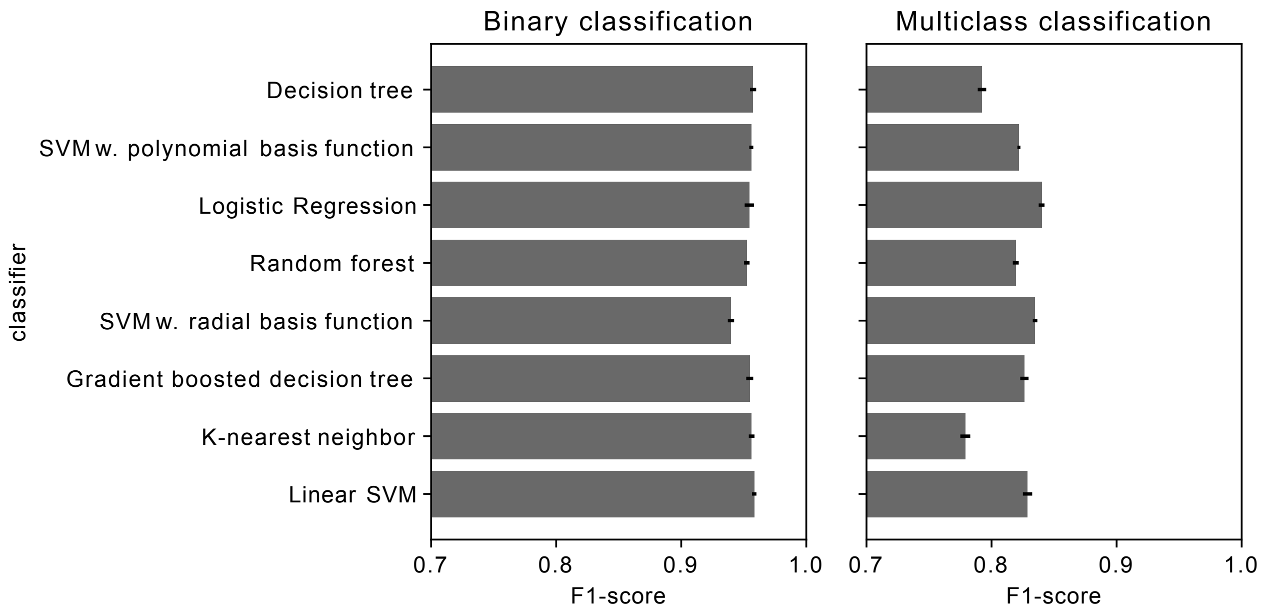

Fig. S1. F1 score for 8 classifiers on the Slow-fast data set presented in Fig. 2 (Binary classification) and the classification data set used in Fig. S11 (Multiclass classification). All models are from the sklearn library. Parameter grid searches were conducted for the Decision tree, all svm classifiers and the nearest neighbor algorithm. For the SVMs, C=(0.1, 1, 10, 100, 1000) was searched, for the decision tree max_depth=(4, 16 , 64, None), min_samples_split=(1, 2, 4, 6), min_samples_leaf=(2, 8, 16, 32) was searched, and for nearest neighbor n_neighbors=(2,8,32,128) was searched. Data was scaled to 0 mean and unit variance for all classifiers except the decision-tree based classifiers (Decision tree, Random forest, and Gradient boosted decision tree). 70% of the data was used for training and 30% for validation.

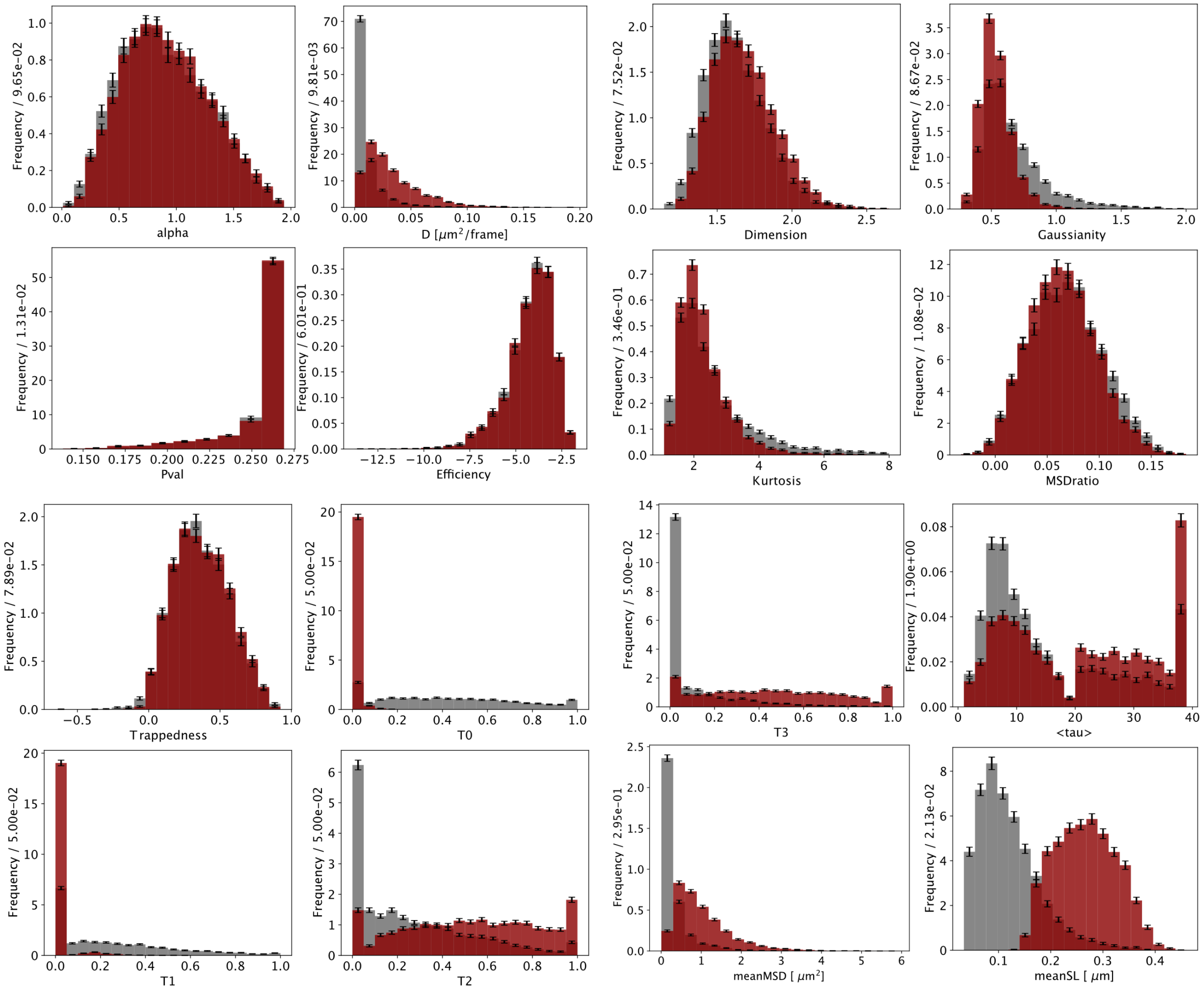

Fig. S2. Feature distributions for the Slow (grey) and Fast (red) simulated data shown in Fig. 2, A, B, C, D of the main text. The feature N (duration of the trace) is not shown as all traces had the same length of 40 frames.

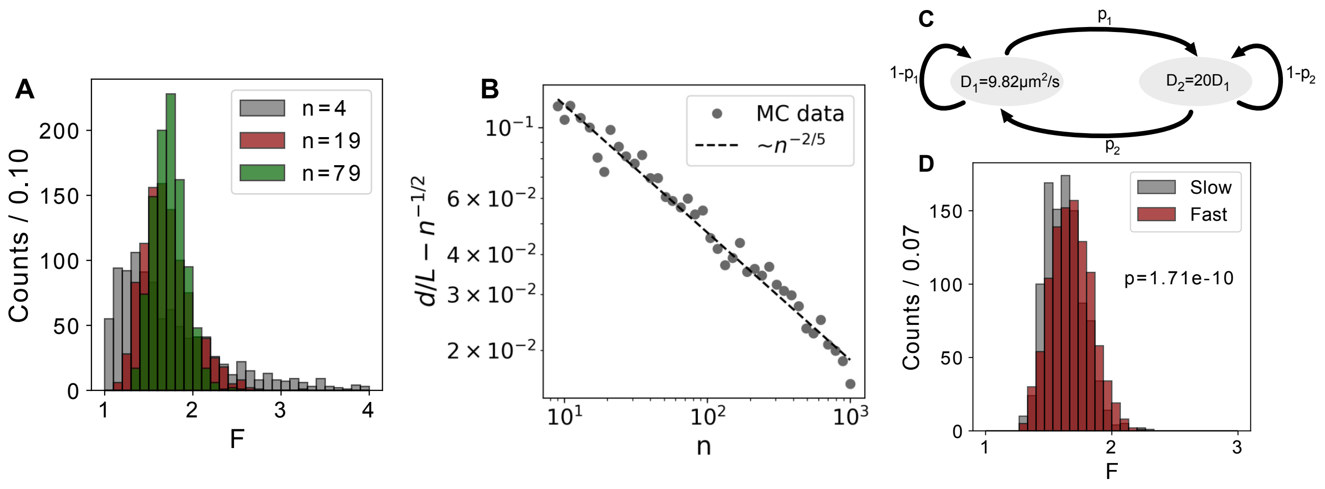

Fig. S3. Simulation investigation on the distribution of estimated fractal dimensions. A: Normal diffusion with varying trajectory lengths. Each distribution includes fractal dimensions estimated for 5000 traces. Note that all distributions are peaked around a value distinctly less than two which is the true fractal dimension for normal diffusion. B: Asymptotic approach of the estimated d/L towards the theoretically expected value of $n^{-1/2}$. The convergence is slightly slower than $n^{-1/2}$ usually seen for estimator variances. C: HMM model used to generate panel D. Two normal diffusion states where used where state 2 has a diffusion constant 20 times that of state 1. p_1 and p_2 refer to transition probabilities away from state 1 and 2 respectively. D: Estimated fractal dimensions for a slow diffuser $(p_1 = 0.05, p_2 = 0.1)$ and a fast diffuser$(p_1 = 0.1, p_2 = 0.05)$. The slow variant is seen to have a distribution for estimated fractal dimension significantly lower than the fast variant (the p-value refers to a Kulmorogov-Smirnoff test). Each distribution contains fractal dimensions estimated over 5000 traces.

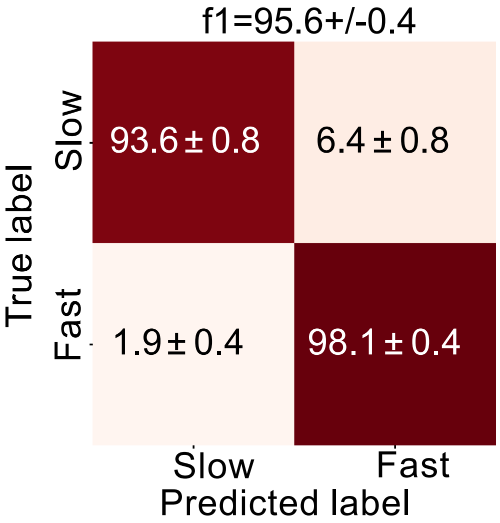

Fig. S4. Training a Logistic Regression classifier using only the top four ranking features does not alter the classification accuracy. Confusion matrix for 5-fold stratified cross validation with a logistic regression classifier trained only on the top four highest ranking features: T0, meanSL, T2 and T3. 80% of the data was used for training and 20% for prediction. The data sets are the slow and fast simulated variants presented in the Fig. 2: A, B, C, D of the main text.

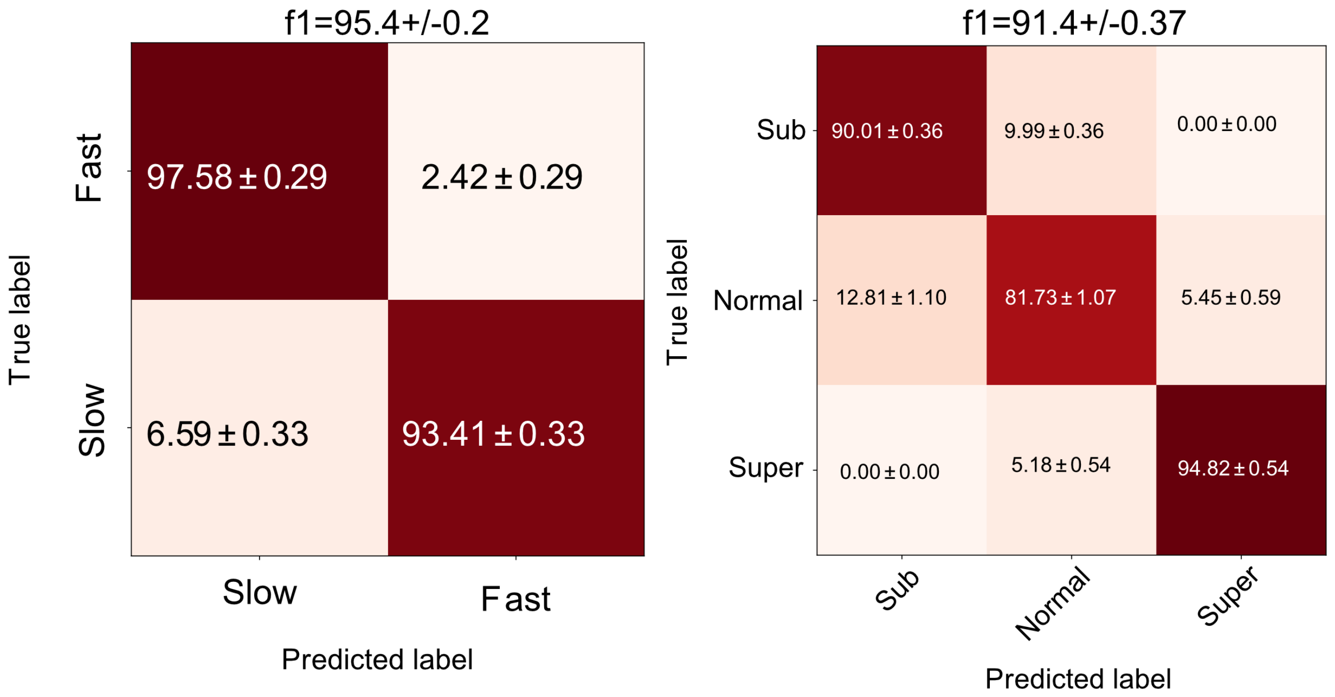

Fig. S5. Confusion matrices for prediction using a Gradient Boosted Decision Tree instead of a Logistic Regression classifier on the data presented in Fig. 2 of the main text.

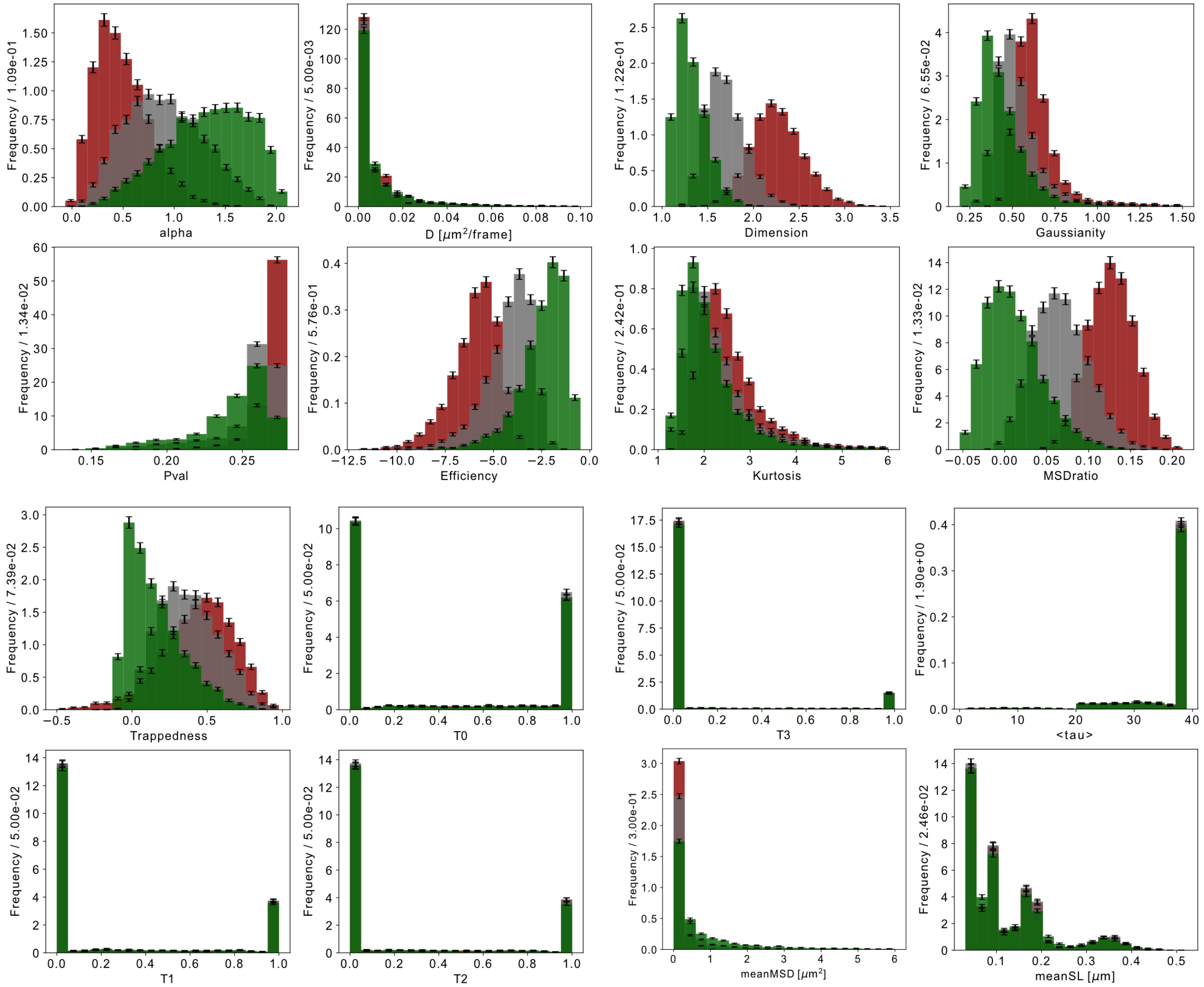

Fig. S6. Distributions for the 17 features output by the diffusional fingerprint for simulated subdiffusion (red), normal diffusion (grey) and superdiffusion (green) presented in Fig. 2: E, F, G, H. Each feature is denoted in the axis and the feature. N (duration of the trace) is not shown as all traces had the same length of 40 frames.

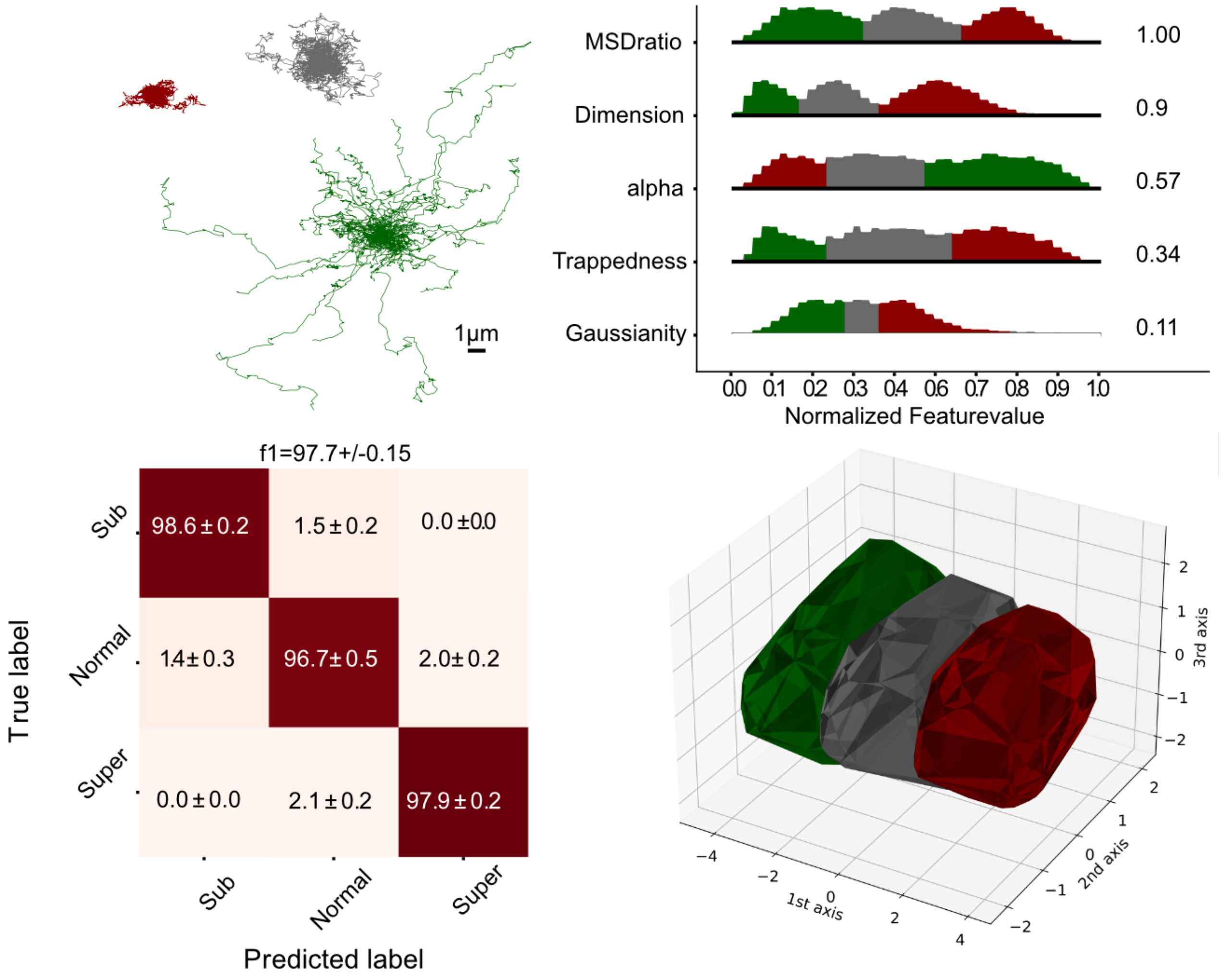

Fig. S7. The fingerprinting classification performance is improved by increased trace length. Shown is the analysis presented for trajectories of varying degrees of straightness (Fig. 2. E, F, G, H) with 100 frames instead of 40 frames. A: Overlaid trajectories of 100 traces for the 3 different condition: Subdiffusive (red), normally diffusing (grey) and superdiffusing (green). B: Distribution of the dominant descriptive features found from LDA projection C: Confusion matrix based on Logistic Regression showing >95% accurcay D: 3D PCA displaying the diffusional map of the fingerprints.

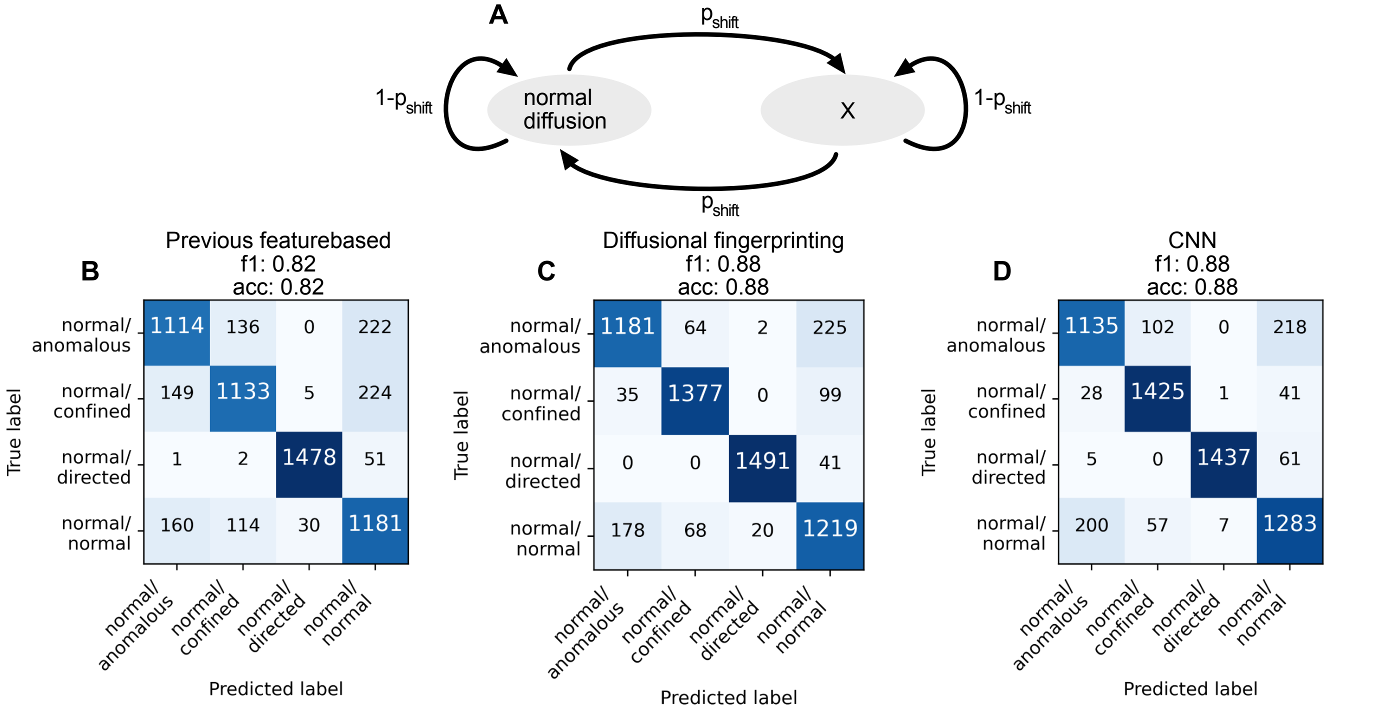

Fig. S8. Comparison with state-of-the art classifiers on a stress-test simulated data set. A: Schematic of the HMM used to generate the data. A two-state HMM was used where the first state is normal diffusion and the other state, X, is either normal diffusion, confined diffusion, directed motion or anomalous diffusion. Simulations where done as described in (1) with parameters: N=100, Δt=1/30 s, D = 9.02 µm^2/s, α=0.3, R=9, B=4, Q=5 and p_{shift} =0.1. A data set of 20,000 traces where generated out of which 70% was used for training and 30% was used for validation. B: Confusion matrix for prediction with the features and gradient boosted decision tree parameters presented in (1). C: Confusion matrix for prediction using diffusional fingerprinting features and the same gradient boosted decision tree as in(1). D: Confusion matrix for prediction using the convolutional neural network (CNN) proposed in (1).

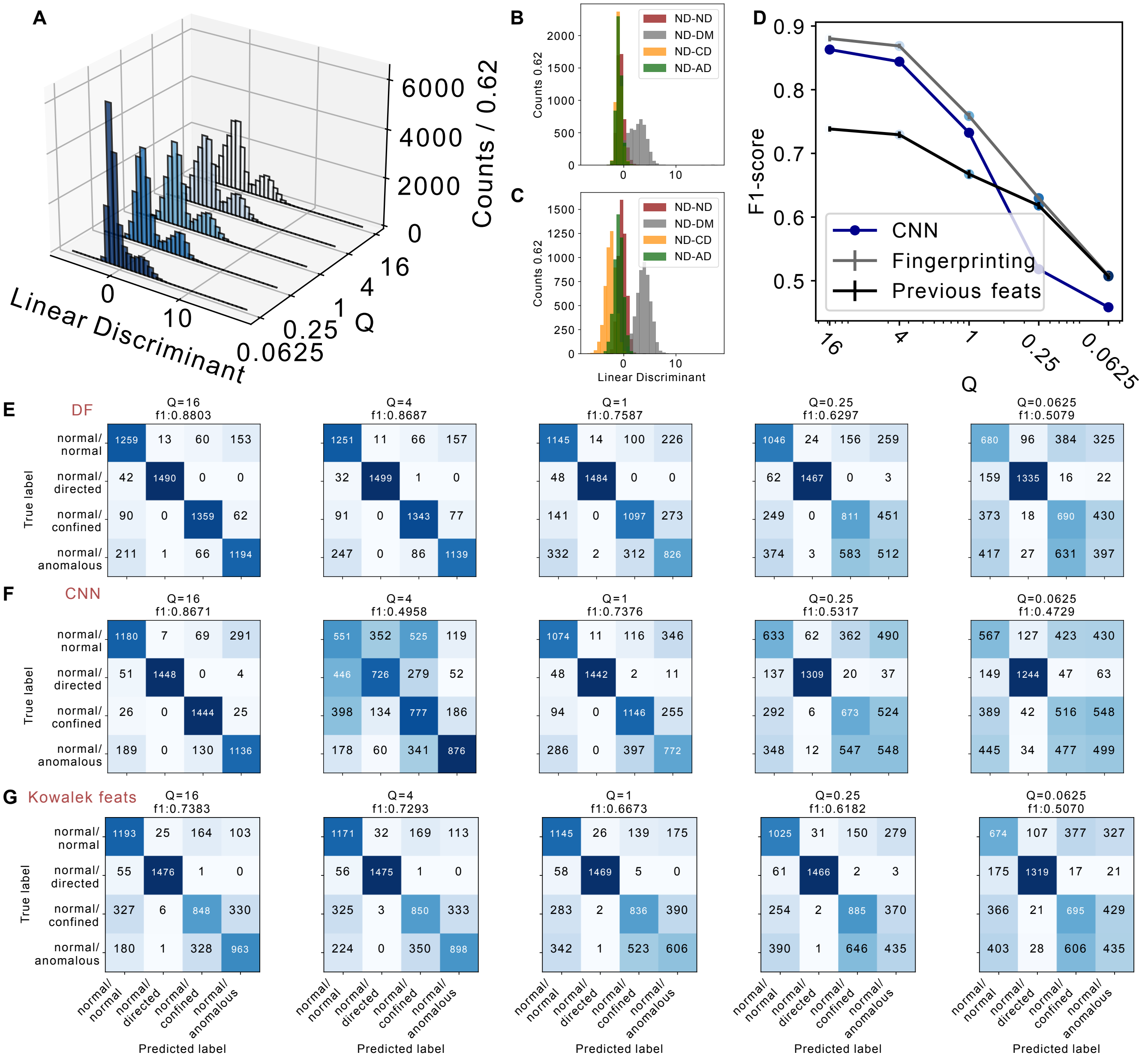

Fig. S9. Quantification of the effects of localization errors on the diffusional fingerprint and comparisons with classifiers used in the field. The simulation is as in S13 but Q is varied (Q is here the ratio of normal diffusion length to localization error: $\sqrt{D\Delta t}/\sigma$ where σ is the standard deviation of the localization error. High Q is low localization error). Abbreviations are as follows: ND-ND:normal/normal, ND-DM: normal/directed, ND-CD: normal/confined and ND-AD: Normal/anomalous A: 1D LDA projection histograms of all the data at various Q. B: 1D LDA projection of the four classes at $Q = 0.0625$. Note that all classes are highly overlapping except ND-DM which displays dynamics not blurred out by the localization error. C: 1D LDA projection of the four classes at $Q = 16$. Note that the ND-CD are now also clearly discernible but the intrinsic overlap between ND-AD and ND-ND persists. D: F1-score for a classification task on 30% of data with a logistic classifier trained on 70% of the data (only for feature-based classifiers). Colors match those of A. Error bars are standard deviations across a five-fold cross validation. E: Confusion matrices for diffusional fingerprinting features using a logistic regression across the 5 data sets. F: Confusion matrices for predictions with the CNN proposed by Kowalek *et al.* (1). G: Confusion matrices for predictions with the features proposed by Kowalek *et al.* using a logistic regression classifier. Usually one would probably only analyze data with $Q > 1$ as for $Q < 1$ the trajectories would be dominated by noise, requiring very long trajectories to identify any underlying dynamics.

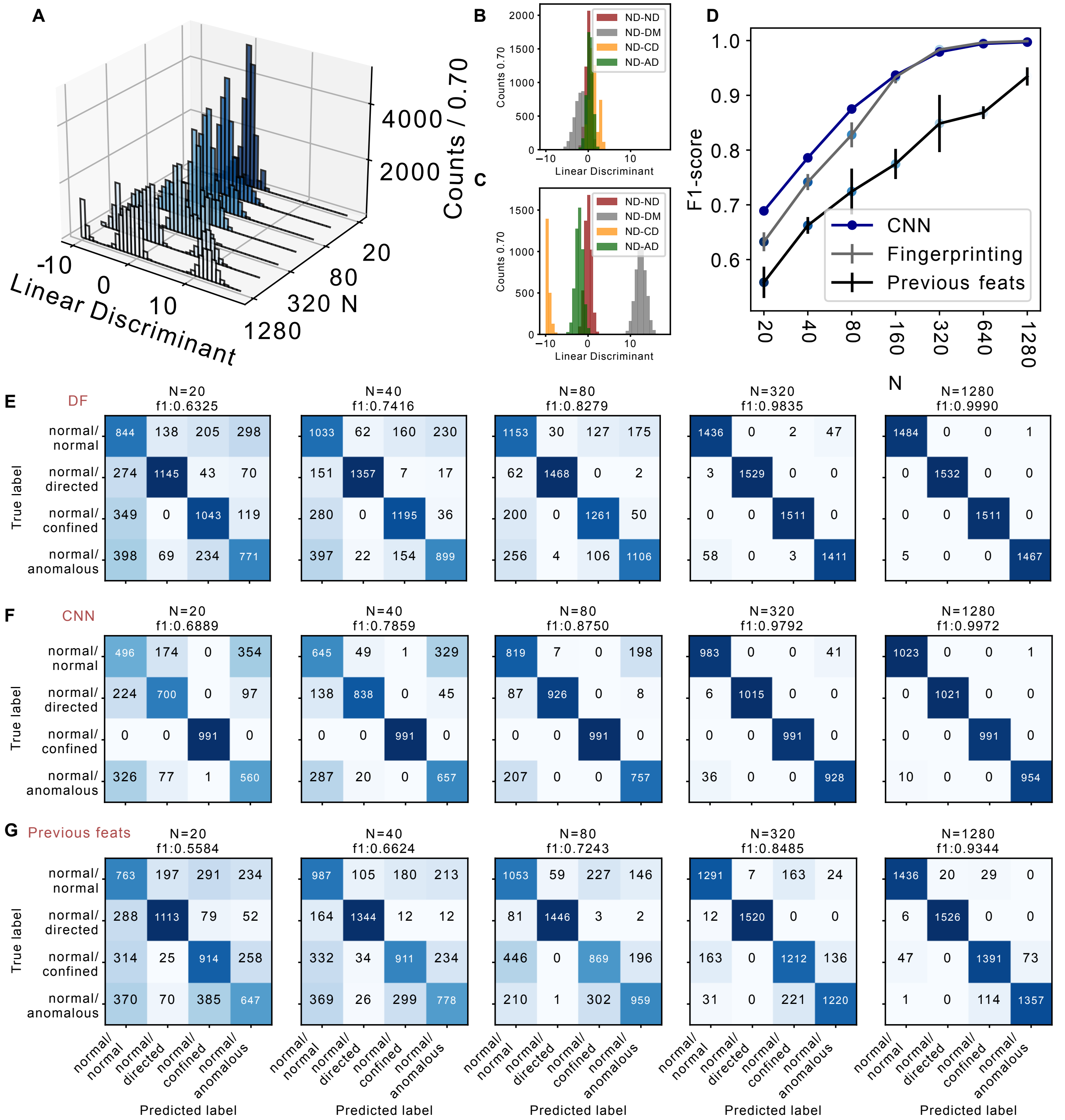

Fig. S10. Quantification of the effects of low signal to noise and high density leading to shorter trajectories. Comparison is done on the diffusional fingerprints along with classifiers used in the field. The simulation is as in S13 but N is varied (N is the trace duration). Abbreviations are as follows: ND-ND:normal/normal, ND-DM: normal/directed, ND-CD: normal/confined and ND-AD: Normal/anomalous A: 1D LDA projection histograms of all the data at various N. B: 1D LDA projection of the four classes at $N = 5$. Note that all classes are highly overlapping. C: 1D LDA projection of the four classes at $N = 1280$. Note that the ND-CD are now also clearly discernible but the intrinsic overlap between ND-AD and ND-ND persists. D: F1-score for a classification task on 30% of data with a logistic classifier trained on 70% of the data (only for feature-based classifiers). Colors match those of A. Error bars are standard deviations across a five-fold cross validation. E: Confusion matrices for diffusional fingerprinting features using a logistic regression across the 5 data sets. F: Confusion matrices for predictions with the CNN proposed by Kowalek *et al*(1). G: Confusion matrices for predictions with the features proposed by Kowalek *et al.* using a logistic regression classifier.

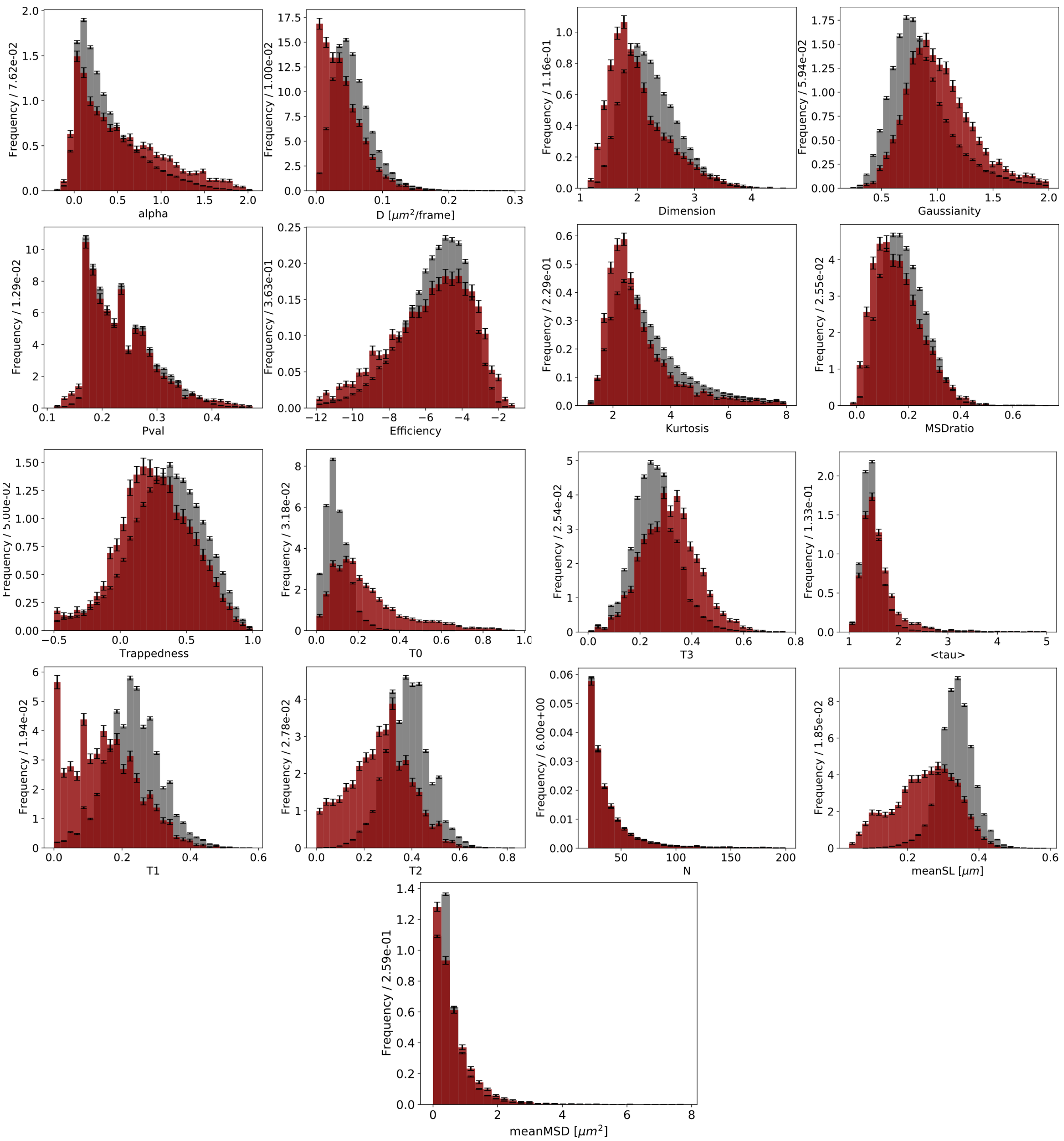

Fig. S11. Overview of the 17 features output by the diffusional fingerprinting for native TLL (red) and L3 (grey). Each feature is denoted in the axis.

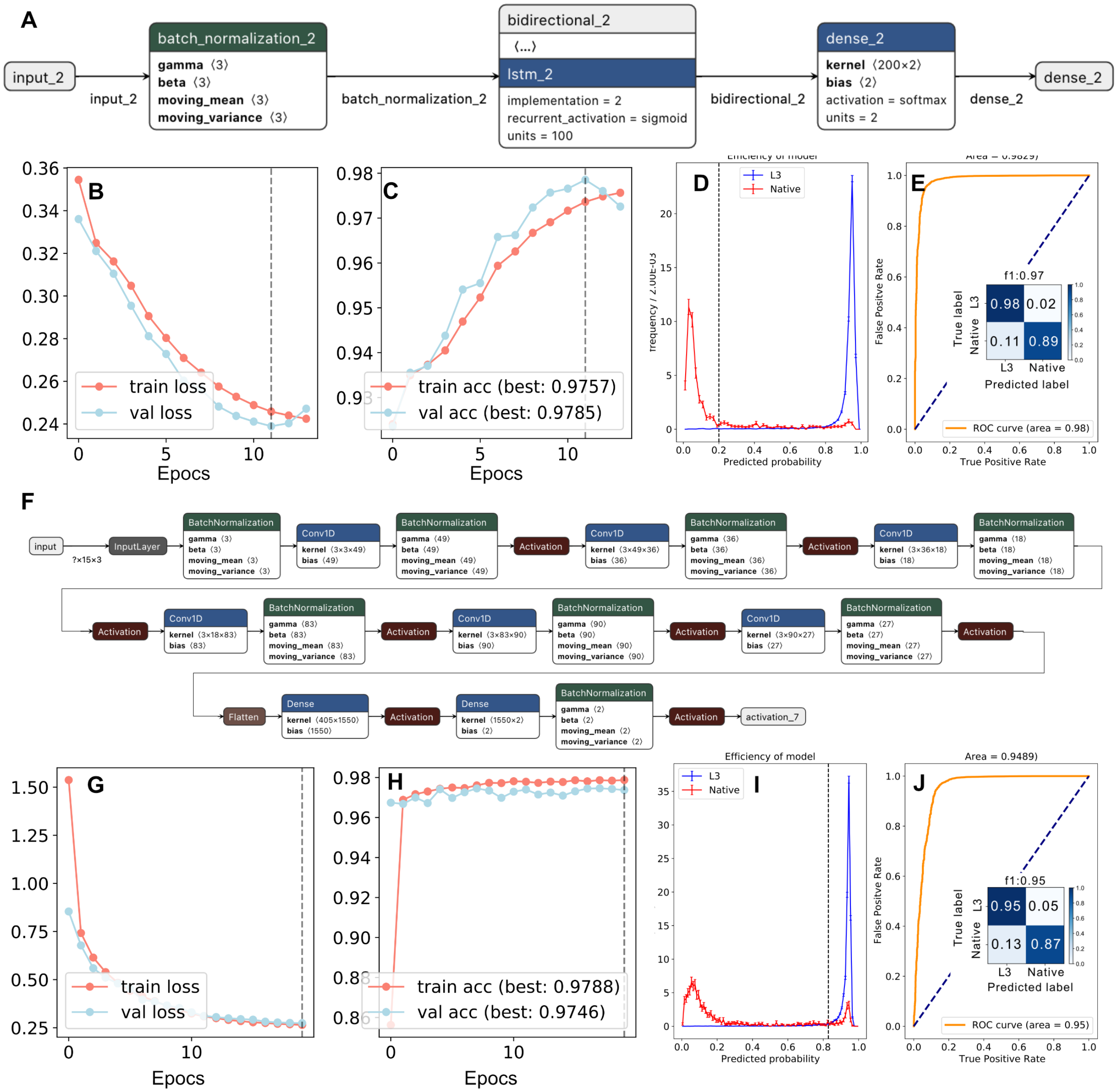

Fig. S12. LSTM neural network trained on x,y and step length data for L3-Native classification using a data set of 5630 traces from each class for a balanced data set (there are 68200 L3 traces and 5630 Native traces in the original data). Training was done on 80% of the data and testing was done on 20%. A: A schematic of the architecture used. B: Learning curve displaying training loss over time, reaching stability over 20 epochs C: Training accuracy, dashed line indicates the chosen model. D: Histogram of output neuron activation on the training data. The dotted line denotes the chosen decision boundary used in the final model. E: ROC curve for classification on the training data. Inset shows confusion matrix for prediction on the test data with the trained model. F,G,H,I,J. Same as for the LSTM but using the convolutional neural network proposed in (1).

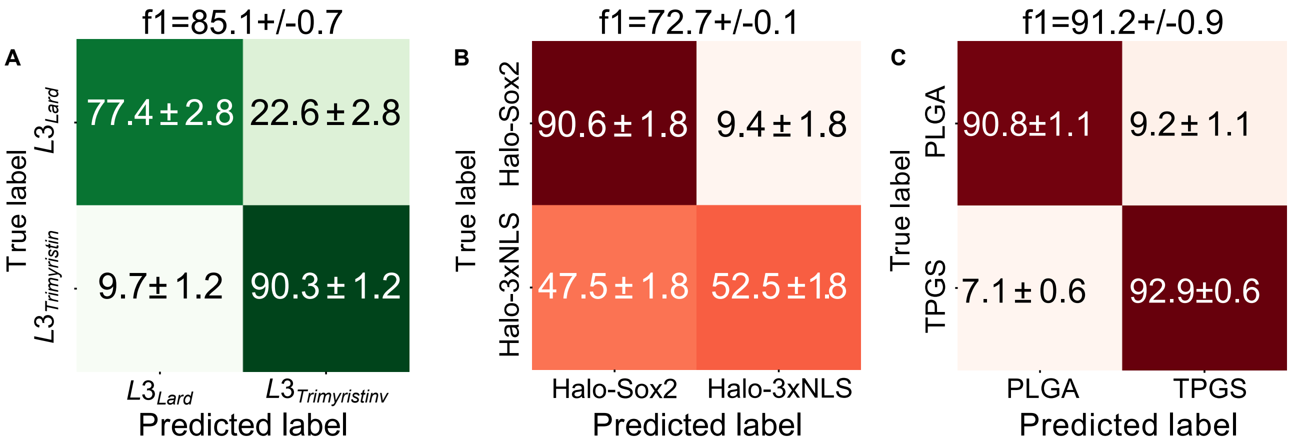

Fig. S13. Confusion matrix for 5-fold stratified cross validation with a Logistic Regression classifier trained on 80% of the data and predicting on 20% of the data. The data sets are those from Fig. 4 in the main text, A is the Lard-trimyristin data presented in Fig. 4: A,B,C, B is the transcription factor data presented in Fig. 4: D,E,F and finally C is the nanoparticle data presented in Fig. 4: G,H,I.

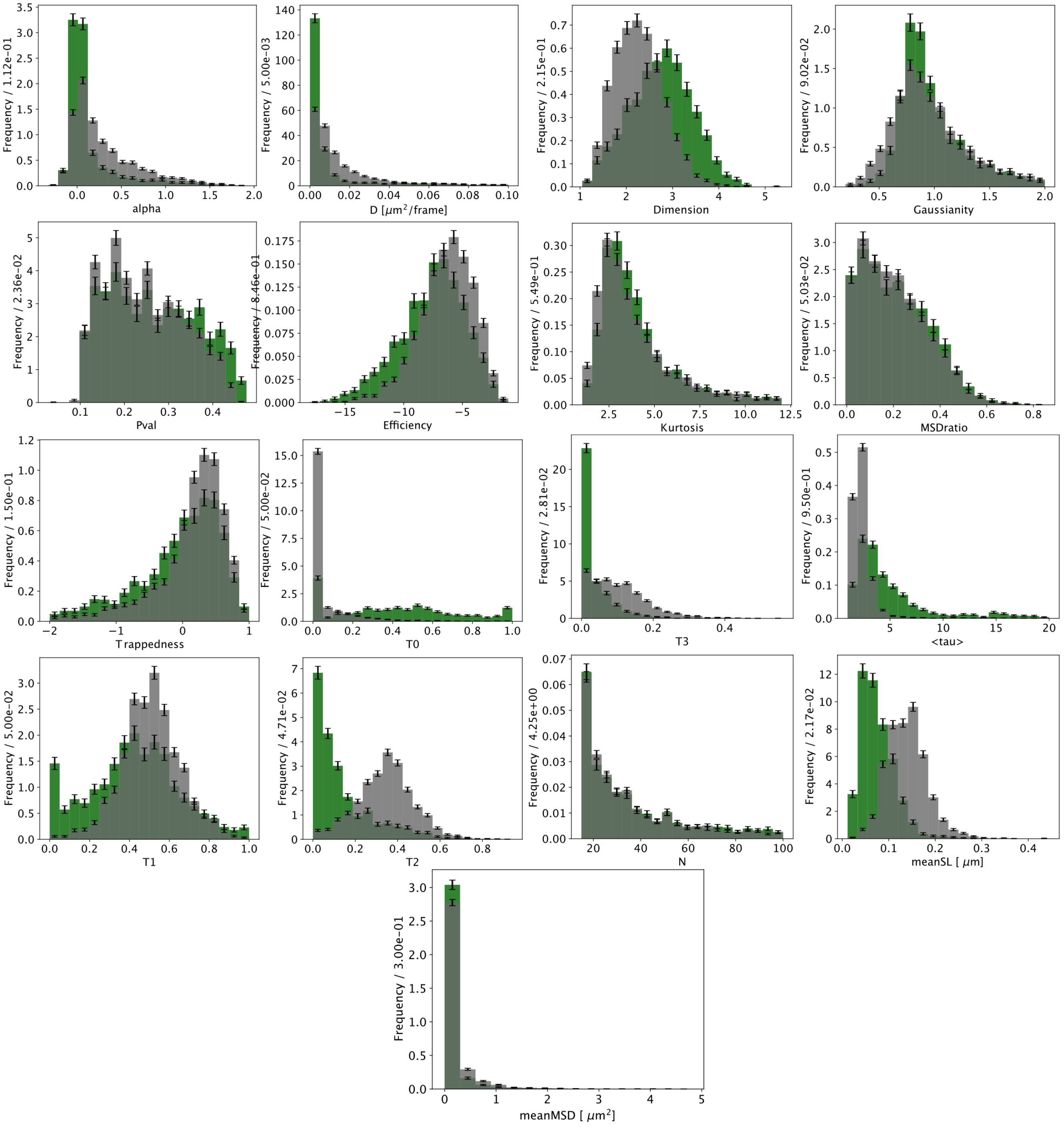

Fig. S14. Feature distributions for the data for lipases on different substrate used for the first column in Fig.4 in the main text. Colors are as in the main text: green: L3 on lard, grey: L3 on trimyristin

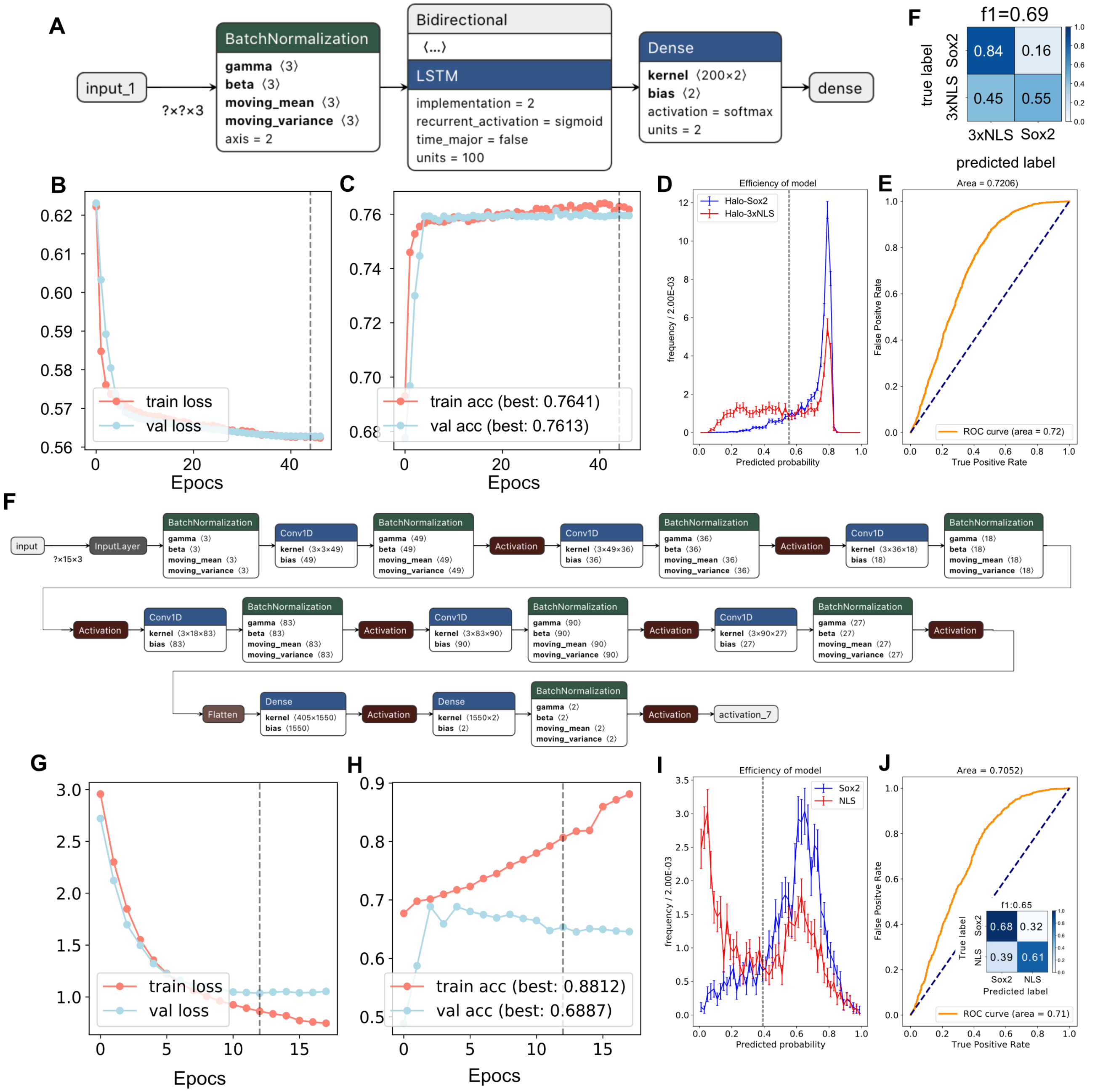

Fig. S15. Bidirectional LSTM trained on the raw step length and x,y-coordinates and a CNN trained on the x,y-coordinates. The data used is the transcription factor data presented in Fig. 4. D,E,F of the main text. A: Graphical schematic of the trained architecture. B: Loss as a function of epochs in the stochastic gradient descent training. C: Accuracy for the same epochs as in B. D: Predicted probability histogram on the validation set with a cutoff that maximizes the sum of specificity and sensitivity marked with a black dotted line. E: ROC curve computed for the bins in the left histogram. F: Confusion matrix for prediction with the cutoff marked in D on the validation data set. F, G, H, I, J: Same as for the LSTM but using the CNN suggested in (1)

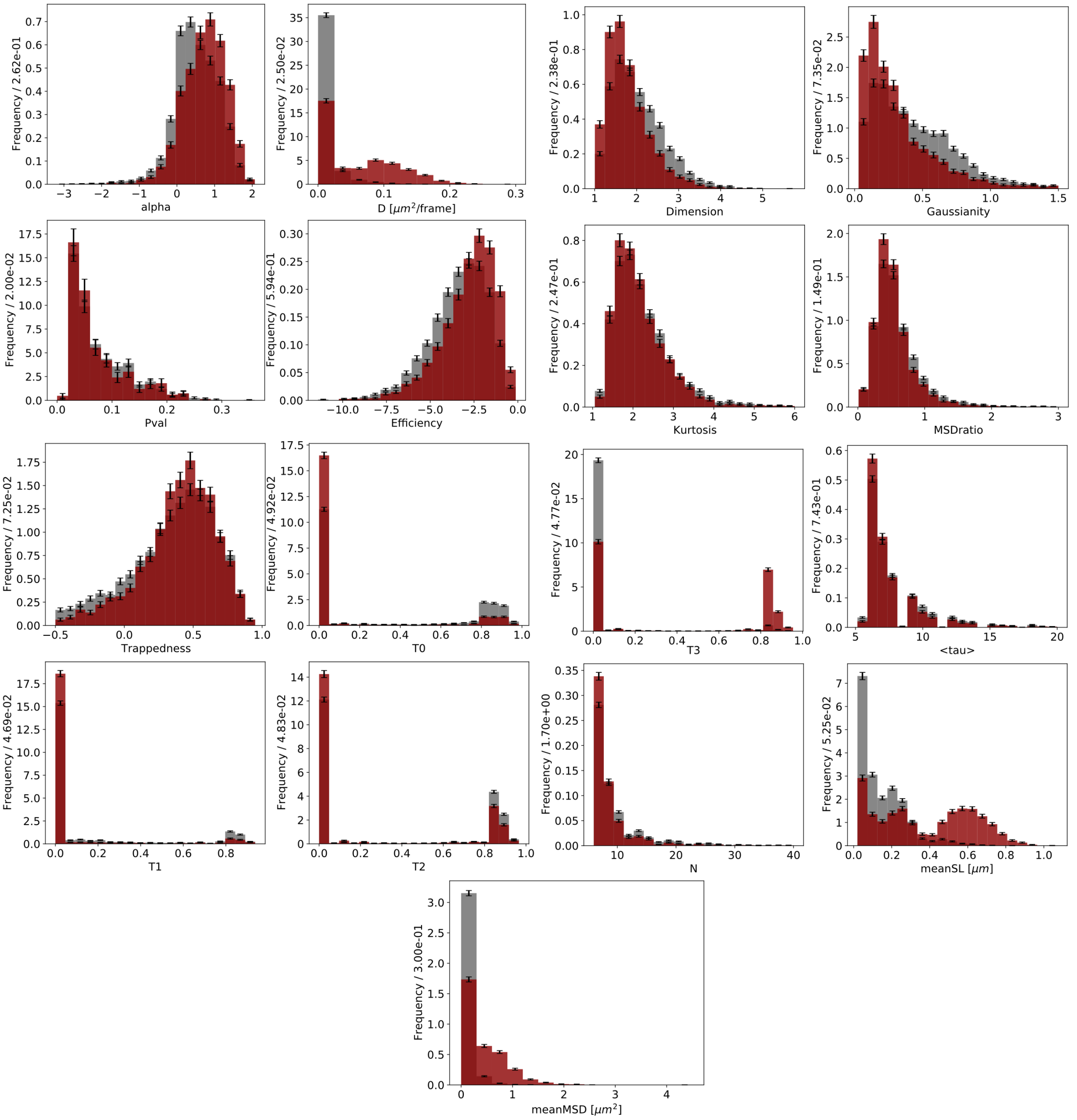

Fig. S16. Feature distributions for the 17 features output by the diffusional fingerprint for the transcription factor data used Fig. 4: D, E, F in the main text. Colors are as in the main text: red: Halo-3xNLS, grey: Halo-Sox2 and each feature is denoted in the axis. Note on the agreement between the results of Hansen *et al.*: The meanSL histogram displays two peaks for NLS and one peak for Sox2 with a tail. Both Sox2 and NLS has a peak from 0-0.25 µm. The NLS peak lies a little below 0.6 µm and is rather broad and the tail for Sox2 could be reconcilable with a similarly broad distribution centered around 0.3-0.4 µm. Since the data is measured with a framerate of 134 Hz, the conversion from meanSL to diffusion constant is D =meanSL$^2/(4/134)$ leading to $D \in \{$0 µm^2/s, 2.1 µm^2/s$\}$ for the shared peak, $D \in \{$3 µm^2/s, 5.4 µm^2/s$\}$ and D =12 µm^2/ for the NLS peak. Hansen *et al.* found a free diffusion constant for Sox2 of roughly 3 µm^2/s, for NLS it was 12 µm^2/s . The positions of the peaks therefore match rather well if one assumes the shared peak from 0-3µm in meanSL is due to bound diffusion.

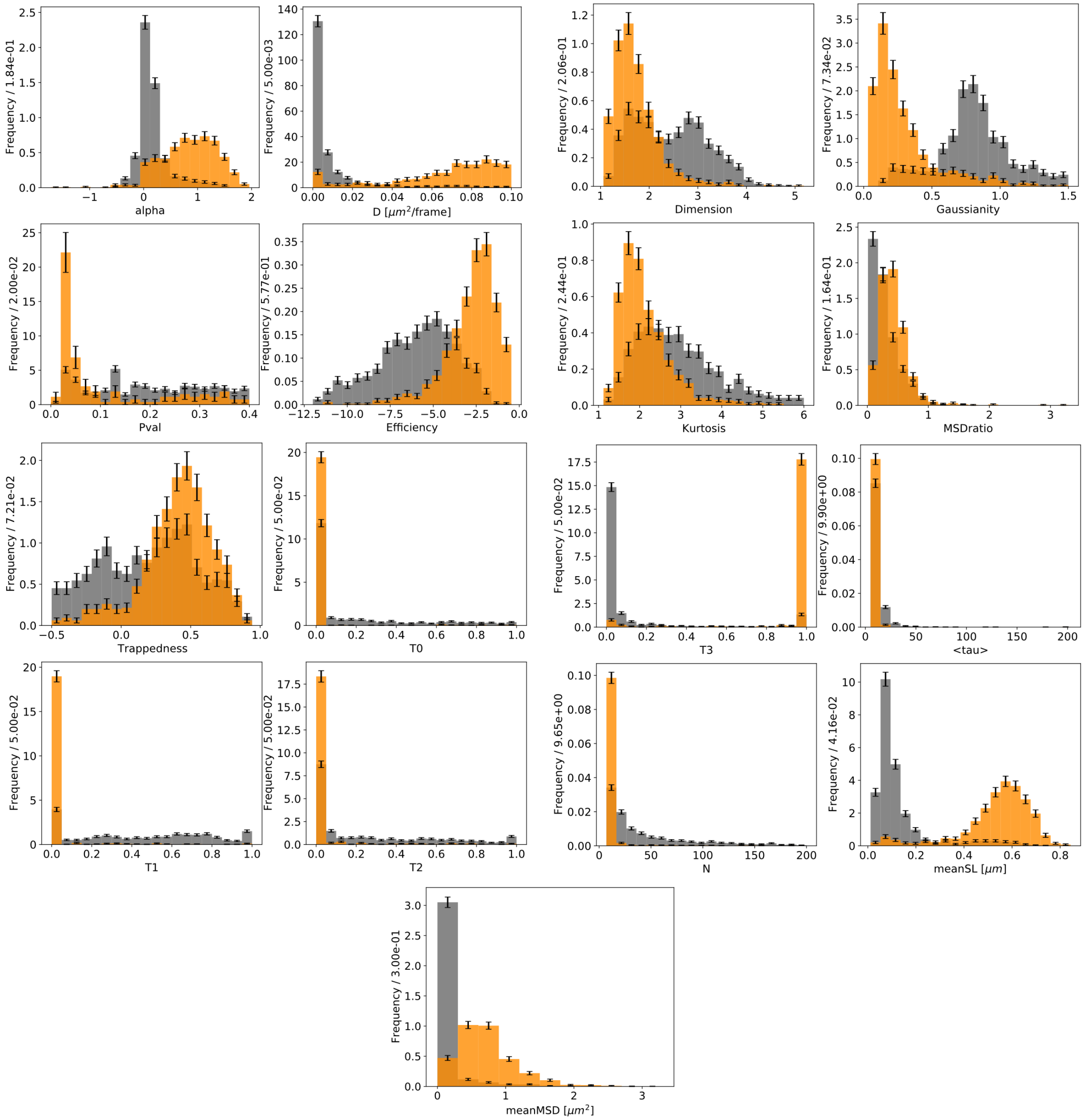

Fig. S17. Distributions for the 17 features output by the diffusional fingerprint for the two nano-particles: PLGA (grey) and TPGS (orange) presented in Fig. 4: G, H, I. Each feature is denoted in the axis.

References

1. P Kowalek, H Loch-Olszewska, J Szwabiński, Classification of diffusion modes in single-particle tracking data: Feature-based versus deep-learning approach. *Phys. review. E* **100**, 032410 (2019).
2. AS Hansen, et al., Robust model-based analysis of single-particle tracking experiments with spot-on. *eLife* **7** (2018).
3. D Ernst, J Köhler, Measuring a diffusion coefficient by single-particle tracking: statistical analysis of experimental mean squared displacement curves. *Phys. Chem. Chem. Phys.* **15**, 845–849 (2013).
4. MJ Saxton, Lateral diffusion in an archipelago. single-particle diffusion. *Biophys. J.* **64**, 1766–1780 (1993).
5. D Ernst, J Köhler, M Weiss, Probing the type of anomalous diffusion with single-particle tracking. *Phys. Chem. Chem. Phys.* **16**, 7686–7691 (2014).
6. MJ Katz, EB George, Fractals and the analysis of growth paths. *Bull. Math. Biol.* **47**, 273–286 (1985).
7. SSR Bohr, et al., Direct observation of thermomyces lanuginosus lipase diffusional states by single particle tracking and their remodeling by mutations and inhibition. *Sci. Reports* **9**, 16169 (2019).
